# 3D interaction homology: The hydrophobic residues alanine, isoleucine, leucine, proline and valine play different structural roles in soluble and membrane proteins

**DOI:** 10.3389/fmolb.2023.1116868

**Published:** 2023-03-28

**Authors:** Mohammed H. AL Mughram, Claudio Catalano, Noah B. Herrington, Martin K. Safo, Glen E. Kellogg

**Affiliations:** ^1^ Department of Medicinal Chemistry and the Institute for Structural Biology, Drug Discovery and Development, Virginia Commonwealth University, Richmond, VA, United States; ^2^ Center for the Study of Biological Complexity, Virginia Commonwealth University, Richmond, VA, United States

**Keywords:** hydrophobic residues, hydropathic interactions, solvent-accessible surface area (SASA), membrane proteins, lipid interactions

## Abstract

The aliphatic hydrophobic amino acid residues—alanine, isoleucine, leucine, proline and valine—are among the most common found in proteins. Their structural role in proteins is seemingly obvious: engage in hydrophobic interactions to stabilize secondary, and to a lesser extent, tertiary and quaternary structure. However, favorable hydrophobic interactions involving the sidechains of these residue types are generally less significant than the unfavorable set arising from interactions with polar atoms. Importantly, the constellation of interactions between residue sidechains and their environments can be recorded as three-dimensional maps that, in turn, can be clustered. The clustered average map sets compose a library of interaction profiles encoding interaction strengths, interaction types and the optimal 3D position for the interacting partners. This library is backbone angle-dependent and suggests solvent and lipid accessibility for each unique interaction profile. In this work, in addition to analysis of soluble proteins, a large set of membrane proteins that contained optimized artificial lipids were evaluated by parsing the structures into three distinct components: soluble extramembrane domain, lipid facing transmembrane domain, core transmembrane domain. The aliphatic residues were extracted from each of these sets and passed through our calculation protocol. Notable observations include: the roles of aliphatic residues in soluble proteins and in the membrane protein’s soluble domains are nearly identical, although the latter are slightly more solvent accessible; by comparing maps calculated with sidechain-lipid interactions to maps ignoring those interactions, the potential extent of residue-lipid and residue-interactions can be assessed and likely exploited in structure prediction and modeling; amongst these residue types, the levels of lipid engagement show isoleucine as the most engaged, while the other residues are largely interacting with neighboring helical residues.

## Introduction

The structural roles of the amino acid residues within proteins have been studied and debated since even before X-ray diffraction data collected from crystals were painstakingly and laboriously analyzed to ultimately reveal the first actual crystal structures. Linus Pauling proposed the α-helix and β-strand motifs based on his knowledge of chemistry and diffraction patterns (Pauling et al., 1951) before they were actually seen in a protein’s structure. The crystal structures of myoglobin by John Kendrew (Kendrew et al., 1958; Kendrew et al., 1960) and hemoglobin by Max Perutz (Perutz, 1960; Perutz, 1962) largely confirmed Pauling’s hypotheses. Pauling’s original musings on structure were focused on hydrogen bonding, which of course is a very critical component of protein structure, especially amongst the backbone atoms of a chain. The richness of sidechain-sidechain residue-residue interactions, however, was not appreciated until it could be systematized from multiple crystal structures, e.g., in the atlas composed by Juswinder Singh and Janet Thornton (Singh and Thornton, 1992). Perhaps the most fascinating interactions are those that are classified as “hydrophobic”, because their seemingly obvious nature is actually disguising a complex molecular event that involves enthalpy, entropy and solvation components (Spyrakis et al., 2017). The fact that hydrophobic phenomena are inherent in proteins was recognized by Irving Klotz (Klotz, 1958) *before* the first X-ray structures were available. Later, a number of researchers recognized that the hydrophobicity of residue sidechains has a relationship with protein secondary structure, thus igniting a plethora of schemes and algorithms to exploit this observation in protein structure prediction (Simm et al., 2016).

Hydrophobicity as a macro, molecular property has also been studied and reported since the late 1800s (Overton, 1899; Leo et al., 1971). In its most common form, hydrophobicity is taken as the log of the ratio of a molecule’s solubility in 1-octanol and in water, i.e., $\log_{10}\frac{{\left[M\right]}_{1-octanol}}{{\left[M\right]}_{water}}$ , or log P_o/w_. For the purposes of drug discovery and development, log P_o/w_ represents an easy-to-use proxy for lipid and membrane transport to sites of action, e.g., for orally administered drugs. The famous Lipinski “rules of five” (Lipinski et al., 2001) suggest that compounds with log P_o/w_ > 5 may not be orally active. In addition to direct measurement of log P_o/w_, considerable effort has been expended in developing prediction algorithms, with hundreds of articles and dozens of reviews or benchmarking studies (Buchwald and Bodor, 1998; Grassi et al., 2002; Machatha and Yalkowsky, 2005; Aliagas et al., 2022).

These two somewhat different views of the same phenomenon, hydrophobicity, coalesced in the mind of Donald J. Abraham, whom this article collection is memorializing. Abraham was a medicinal chemist who realized before virtually all of his colleagues the potential power of using X-ray crystal structures to design drugs. He convinced Max Perutz to let him come to Cambridge and pursue this idea in search of molecules that could modulate hemoglobin, in particular as a treatment for sickle cell disease (Perutz et al., 1986). Also, as a medicinal chemist, Abraham was well aware of the lengthy and expensive process to design and develop a *drug*, so he had a keen interest in computational tools that could facilitate the process, especially in the context of the emerging structure-based paradigms. Another article in the collection reviews the origins and capabilities of our HINT program (Kellogg and Abraham, 2000; Sarkar and Kellogg, 2010), which was thus designed by Abraham and Kellogg to connect the rich information content of log P_o/w_ (from medicinal chemistry) with X-ray crystallographic structural data (from structural biology).

### 3D interaction homology

The focus of this contribution is also on the relationship between hydrophobicity and structure and was inspired by Abraham’s vision. It utilizes a very specific feature and application of HINT. The hypothesis is that it is the character of residues, and in particular, their three-dimensional interaction networks that drive protein structure on multiple scales. This rather obvious assertion is in seeming contrast with the dogma of sequence homology being the key factor in protein folding, *etc.* In actuality, these two notions merge in cases of higher sequence homology or similarity. In our approach, each residue in a protein has a *hydropathic valence*, which is the constellation of interactions that it ideally would make, including interaction type (e.g., hydrophobic, hydrogen bond, *etc.*), strength of interaction and spatial arrangement of these interactions. Interestingly, we have shown that there are a limited number of these interaction sets, dependent on residue type and backbone angles, and they can be represented as three-dimensional sets of contourable hydropathic interaction maps. In previous publications, as we developed this paradigm, these results were demonstrated for a number of residue types: tyrosine (Ahmed et al., 2015), alanine (Ahmed et al., 2019), phenylalanine, tyrosine and tryptophan (Al Mughram et al., 2021a), serine and cysteine (Catalano et al., 2021), and aspartic acid, glutamic acid and histidine (Herrington and Kellogg, 2021). Further, these studies illustrated that the observed 3D map profiles are conserved motifs (Ahmed et al., 2019), the hydropathic interaction maps carry even subtle interaction information like pi-pi stacking and pi-cation interactions (Al Mughram et al., 2021a), have scope for adjustable pH (Herrington and Kellogg, 2021), and provide insight into the formation of cysteine-cysteine bridges (Catalano et al., 2021). Finally, another study—a preliminary assessment of the differences between residues in soluble and membrane proteins with regard to their populations, hydropathic interaction characteristics and solvent-accessible surface areas as functions of backbone conformation (AL Mughram et al., 2021b)—compelled further and deeper investigation into multiple observations from that report.

In this contribution, we focus on the aliphatic hydrophobic residues: alanine, isoleucine, leucine, proline and valine. While the interaction characteristics of the sidechains with their environments are limited to hydrophobic-hydrophobic and hydrophobic-polar types, which can be thought of as *favorable* and *unfavorable* hydrophobic interactions, respectively, the detailed hydropathic interaction map calculations we performed again reveal sets of these that are dependent on the underlying backbone angles (i.e., secondary structure). Furthermore, the solvent-accessible surface areas of these residues (Fraczkiewicz and Braun, 1998), although they are generally fairly “buried”, show backbone angle dependence.

### Membrane proteins

We also evaluate in this work a second dataset, of membrane proteins, where we might expect the interaction roles of these residues to be largely reversed. In other words, while the aliphatic hydrophobic residues are generally buried with low solvent accessibility in soluble proteins, these residues should be “exposed” when embedded within the membrane and available for interaction with the lipid “solvent”. However, membrane proteins consist of multiple components, each with unique characteristics: some residues, e.g., in intracellular and extracellular loops, likely do not interact at all with the lipids; another set of residues with likely minimal direct lipid interactions are those at the core of the trans-membrane region, e.g., in channels or GPCR binding sites; and lastly, a set consisting of the residues that do interact with the membrane/lipids.

Importantly, most reported X-ray crystallographic and cryo-electron microscopic structures of membrane proteins do not contain native-like lipids due to a plethora of issues in extracting and preserving them throughout the measurements (Carpenter et al., 2008; Matar-Merheb et al., 2011; Hendrickson, 2016). A key issue is that detergents are usually used to separate the protein from the membrane, and that procedure can be deleterious to the delicate environment surrounding the protein and facilitating its structure and function (Yang et al., 2014; Chipot et al., 2018; Guo, 2020; Guo, 2021). Thus, computational approaches to evaluate lipid-protein interactions are particularly necessary in order to really appreciate membrane protein structure and function. The basis of our approach, the water-to-octanol partition coefficient, has been shown to be relevant for understanding amino acid sidechains partitioning into lipid bilayers (MacCallum et al., 2007).

To perform this work, we applied several filters from the MemProtMD database (Newport et al., 2019) to populate and characterize the three sets of residue environments in membrane proteins defined above. MemProtMD is a database (http://memprotmd.bioch.ox.ac.uk) of over 5,000 intrinsic membrane protein structures abstracted from the Protein Data Bank, pre-oriented such that the transmembrane axes correspond to z, and inserted into simulated lipid bilayers (dipalmytoylphosphatidylcholine, DPPC), through application of Coarse-Grained Self Assembly Molecular Dynamics simulations. While some PDB-deposited, fully experimentally-derived, membrane protein structures do possess lipid electron density and fitted lipid coordinates, such structures are of inconsistent completeness and quality. To obtain residue-level solvent-accessible surface areas (SASA), we used the GETAREA (Fraczkiewicz and Braun, 1998) algorithm and output. We also adapted GETAREA to define a new parameter, lipid-accessible surface area (LASA); in other words, treating the lipid bilayer as a solvent (McIntosh and Simon, 2007; White, 2007).

### Objectives

With this extensive collection of data in hand, we set out to explore several questions, such as: 1) What are the roles of the aliphatic hydrophobic residues in protein structure and are these roles backbone angle dependent; 2) Are the hydrophobic residues in the extracellular/intracellular data sets from membrane proteins similar to those in the soluble protein set, in terms of residue population frequency and hydropathic character? 3) What are the similarities and differences between the “core” and lipid-facing residues in the transmembrane regions? 4) Are there identifiable and calculable markers in the hydropathic residue interaction maps and derived parameters that may predict the likelihood of a specific residue being in a membrane environment or elsewhere in a protein?

Long range, our vision is to exploit the maps and their associated characteristics for all residues in protein structure prediction settings such as sidechain rotamer optimization, protein-protein docking and *de novo* structure prediction. The prerequisite for that, however, has been building an understanding of the actual roles that each residue type plays in structure. The articles in this series, as referenced above, combined with the new results here for the aliphatic hydrophobic residues, including emerging information about those in membrane proteins, are getting us close to this goal.

## Materials and methods

### Soluble protein dataset

From a collection of 2,703 randomly selected proteins from the RCSB Protein Data Bank, using only structures containing no ligand or cofactor, we extracted all alanine, isoleucine, leucine, proline and valine residues from each structure, excluding N- and C-terminal residues. We have previously described our selection criteria for this protein structure dataset (Ahmed et al., 2015), i.e., to abide by random population-based sampling of a variety of primary, secondary, and tertiary structures. We do not *a priori* exclude proteins with similar or identical sequences, but do believe the size of our dataset likely includes virtually all unique residue environments of alanine, isoleucine, leucine, proline and valine. For similar reasons, we did not apply any resolution cut-offs so that more rare interaction environments that might be present in low-resolution structures would be included. Hydrogen atoms were added to heavy atoms of all structures based on their hybridization, which was followed by conjugate gradient minimizations of their positions using Sybyl X.2.1 (Tripos, St. Louis, MO, United States). Residues from this data set are designated as ALA, ILE, LEU, PRO and VAL.

### Membrane protein dataset

Similarly, we extracted all alanine, isoleucine, leucine, proline and valine residues from 362 membrane protein structures in the Grazhdankin et al. (2020) dataset, which is a subset of the MemProtMD database (Newport et al., 2019) of structures that were, as deposited, pre-oriented, lipid “solvated” and subjected to ∼1 μs of coarse grain molecular dynamics (Stansfeld et al., 2015). In previous work (AL Mughram et al., 2021b; Catalano et al., 2021), our data set was slightly larger, but supplementary files (*vide infra*) for seven proteins (pdbids: 3fb5, 3wxv, 4xwk, 5f1c, 5jsz, 5llu, 5m94) we had used are not currently available. Lipids more than 6 Å away from the protein were removed and missing hydrogen atoms were again added to heavy atoms and energy minimized as above. The MemProtMD dataset structures do not include water molecules, ions or other cofactors. To distinguish residues from the membrane protein dataset, we designated these residues as ALAm, ILEm, LEUm, PROm and VALm.

To bin the residues into sets representing their locations within the membrane proteins, we relied on two of the supplementary files in MemProtMD associated with each protein-lipid model. First, the “*Distortions*“ snapshots are PDB-formatted coordinate files (*pdbid*_default_dppc-distortions.pdb) of the average surfaces formed by lipid phosphates “beads” over the final 800 ns of simulation time (Newport et al., 2019), which can be interpreted as the extents of the membrane region. We averaged the z-coordinates of these “atoms” in the upper and lower planes, which had a standard deviation of ∼1 Å, and defined all residues where the z-coordinates of all three of their backbone atoms, N, CA and O, are between those bounds to be in the membrane region. Residues not meeting that criteria were assumed to be extra- or intramembrane and placed in “soluble” bins, ALAmS, ILEmS, *etc.* Residues in the membrane region were then further classified using the second MemProtMD “*residue-wise analysis*” file (*pdbid*_default_dppc-by-resid.csv) that reports (true/false) if each residue is a constituent of the pore inner surface. For “true” cases, we placed that residue in “core” bins, e.g., ALAmC, ILEmC, *etc.*, while for “false” cases, the residues were placed in the “lipid” bins ALAmL, ILEmL, *etc.* In order to isolate the contribution of protein-lipid interactions, we also calculated (*vide infra*) map data for this last dataset that ignored interactions with lipids, and we identified these results as ALAmN, ILEmN, *etc.*

### Alignment calculations

**FIGURE 1:**
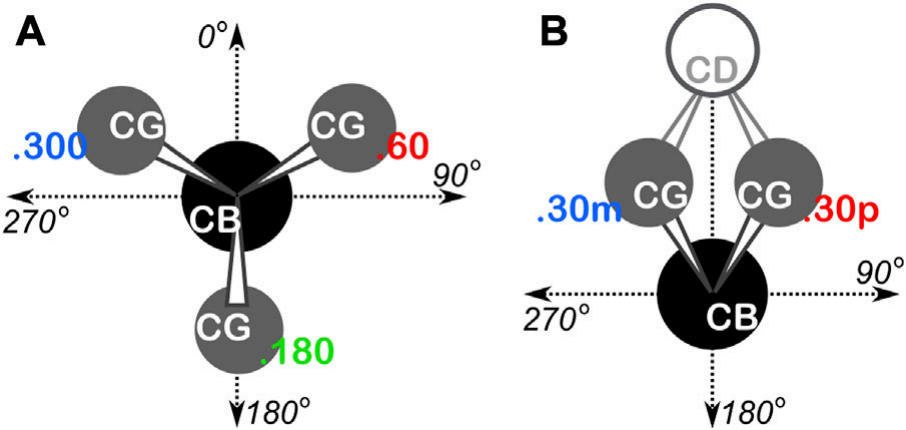
Definition of residue χ_1_ parses. **(A)** Schema used for isoleucine and leucine. The CA atom is behind the CB atom, the torsion is defined as CA-CB-CG-CDn, yielding three rotamers at 60°, 180° and 300°. **(B)** Schema used for proline. The CA atom is behind CB and the torsion is defined as CA-CB-CG-CD, yielding two rotamers at 30° (.30p) and 330° (.30m).

To systematize our analyses with respect to backbone angles, we overlayed an 8 by 8 “chessboard” over the standard plot of Ramachandran ϕ (phi)—ψ (psi) space, with each chess square named ***a1-h8*** and denoted in bold italic (Ahmed et al., 2015). The grids of the boards for alanine, isoleucine, leucine and valine residues were shifted by −20° and −25° in the ϕ and ψ directions, respectively, to optimally position higher-density regions, e.g., to center the highly populated α-helix conformation within a few chess squares. The proline Ramachandran plot’s grid was shifted by −35° and −5° in the ϕ and ψ directions, respectively. The ϕ, ψ, and χ angles were all calculated for every residue in our dataset, and each residue was binned into their proper chess square based on its respective ϕ and ψ angles. All isoleucine and leucine residues in each chess square were further divided by their χ_1_ angles into three parse groups: group “.60” (0° ≤ *χ*_1_ < 120°), group “.180” (120° ≤ *χ*_1_ < 240°), and group “.300” (240° ≤ *χ*_1_ < 360°). In the case of proline, residues were parsed by their χ_1_ angles into two bins, −30° (330°) and +30°, which we will denote as “.30 m” and “.30p”, respectively. See Figure 1 for a schematic of these definitions. These parses were added as suffix to each chess square name, e.g., as ***b1.180***. Further parsing, e.g., χ_1_ for valine or χ_2_ for isoleucine and leucine, is not necessary because the mapping and clustering (*vide infra*) generally captures those structural differences. Supplementary Tables S1-S5 contain all information for each residue of each type in our two datasets, including their chess squares, parses, PDB IDs, ϕ, ψ and ω torsion angles and atom numbers for the backbone atoms and CB of each residue.

All residues of each type were aligned to a model residue at the center of each chess square, with the Cartesian origin at the CA atom, the CA-CB bond corresponding to the *z*-axis, and the CA-HA bond on the yz-plane (Ahmed et al., 2015). Rotation and translation matrices were determined with least-squares fitting of each residue’s constituent atoms to those of the model. Thus, all calculated maps and environments result from that residue’s interactions, and they can be aligned with all other residues of that type in the chess square.

To simplify nomenclature for the following results and discussion, each studied residue was assigned a number in a list of residues within each chess square or, as needed, χ_1_ parse; e.g., the first alanine in the ***a1*** chess square is 1, the third isoleucine in the ***c5.300*** parse is 3, *etc.* Supporting information Supplementary Tables S1A-D, Supplementary Tables S2A-D, Supplementary Tables S3A-D, Supplementary Tables S4A-D and Supplementary Tables S5A-D, for alanine, isoleucine, leucine, proline and valine, respectively, unpack these codes into the actual pdbid, residue number, *etc.*, From our datasets for the soluble protein (a), soluble domain (b) of the membrane protein, lipid-facing (c) and core (d) transmembrane residues. For example, residue 1 for the ***b1*** chess square of (soluble) valine (Supplementary Table S5A) is Val 46A of protein (pdbid) 1A06.

### HINT scoring function

The HINT forcefield (Sarkar and Kellogg, 2010; Kellogg and Abraham, 2000; Kellogg, G. et al., 1991) was used for interatomic interaction scoring. Atom-focused parameters, the hydrophobic atom constant, an atom-level logP_o/w_ (*a*_1_, *a*_*i*_ > 0 for hydrophobic atoms and *a*_*i*_ < 0 for polar atoms), calculated using an approach similar to CLOG-P in that it uses the defined fragments and factors of the Hansch and Leo methodology (Hansch and Leo, 1979; Abraham and Leo, 1987), and solvent-accessible surface area (SASA, *S*_*i*_), calculated from local geometry (Kellogg, et al., 1992), for atom *i*. carry the interaction information.

The interaction score between atoms *i* and *j*, *b*_*ij*_, is calculated by:

$$b_{ij}=a_{i}\;S_{i}\;a_{j}\;S_{j}\;T_{ij}\;e^{-r}+L_{ij},$$

where r is the distance (Â) between atoms *i* and *j*. *T*_*ij*_ is −1, 0, or 1 to account for acidic, basic, *etc.*, character of atoms involved and helps assign the proper sign to the interaction score. Finally, L_*ij*_ implements a Lennard-Jones potential function (Kellogg et al., 1991) described previously. In practice, *b*_*ij*_ > 0 for favorable interactions, such as Lewis acid-base and hydrophobic-hydrophobic interactions, and *b*_*ij*_ < 0 for unfavorable interactions, e.g., hydrophobic-polar or Lewis base-base interactions.

Generally, interactions were calculated for the residue of interest only with respect to all other residue types and water, but for the “lipid” datasets (ALAmL, ILEmL, *etc.*) atoms from the DPPC lipid molecules were considered in calculations.

### HINT basis interaction maps

Each residue was placed within a three-dimensional box large enough to accommodate the structure of a residue, plus an additional 5 Å on each dimension. These boxes, based on residue type, are as follows: alanine, −7.5 Å ≤ x ≤ 8.5 Å; −7.5 Å ≤ y ≤ 8.5 Å; −7.5 Å ≤ z ≤ 8.5 Å (35,937 points, 4096 Å^3^); isoleucine and leucine, −9.0 Å ≤ x ≤ 9.0 Å; −9.0 Å ≤ y ≤ 9.0 Å; −7.5 Å ≤ z ≤ 9.5 Å, (47,915 points, 5,508 Å^3^); proline, −9.5 Å ≤ x ≤ 9.5 Å; −9.5 Å ≤ y ≤ 9.5 Å; −7.0 Å ≤ z ≤ 9.0 Å (50,193 points, 5,776 Å^3^); and valine, −8.5 Å ≤ x ≤ 8.5 Å; −8.5 Å ≤ y ≤ 8.5 Å; −7.5 Å ≤ z ≤ 9.5 Å, (42,875 points, 4913 Å^3^); all with a point spacing of 0.5 Å. As described previously (Ahmed et al., 2015), interaction grids representing the 3D interaction space surrounding residues of interest were calculated. Such maps visualize pairwise HINT scores into 3D objects indicating position, intensity, and type of atom-atom interactions between the residue and those neighboring it. Each grid point for a map was calculated with:

$$\rho_{xyz}=\sum b_{ij}\exp(–[{(x\;–\;x_{ij}{)}^{2}\;+\;(y\;–\;y_{ij})}^{2}+{(z\;–\;z_{ij})}^{2}]/\sigma ),$$

where *ρ*_*xyz*_ is the map interaction score at coordinates (*x*, *y*, *z*), *b*_*ij*_ is the score between atoms *i* and *j*, *x*_*i*j_, *y*_*ij*_ and *z*_*ij*_ are coordinates of the midpoint of the vector between atoms *i* and *j*, and *σ* is the width of the Gaussian map peak, here *σ* = 0.5. Map data were calculated for sidechain atoms of the studied aliphatic hydrophobic residues with individual maps for four interaction classes: favorable polar, unfavorable polar, favorable hydrophobic and unfavorable hydrophobic.

### Calculation of map-map correlation metrics and clustering

The calculations of map-map correlations, i.e., comparisons of two maps, **m** and **n**, was in general terms:

$$if\;\left|G_{t}\right|/F\;>\;1.0,A_{t}=\left(G_{t}/\left|G_{t}\right|\right)\;\log_{10}\;\left(\left|G_{t}\right|/F\right);\;else,A_{t}=0,$$

where each map point (*G*_*t*_, for point at index *t*) is transformed to log_10_ space and normalized with a predefined floor value, F = 1.0. Calculational methods defining the similarity between maps **m** and **n**, defined as *D*(**m,n**) was calculated as described previously in detail (Ahmed et al., 2015). For clustering analysis of the pairwise map similarity matrices, we utilized k-means clustering implemented in the freely available R programming language and environment (R Core Team, 2013). We opted to set a uniform maximum number of clusters of 4 for each chess square of alanine, 9 for each chess square/parse of isoleucine and leucine (up to 27 per chess square), 6 for proline (up to 12 per chess square) and 9 for valine. Thus, we have significant map diversity and scope for inter-chess square/inter-residue comparisons. A limitation of k-means is that it does not form singleton clusters, so we developed protocols to optionally recover them by reconstructing the cluster solutions possessing missing singletons. Any chess square/parse with four or fewer maps was not subjected to clustering, but, was instead averaged to create what is, effectively, a 1-cluster case. Each cluster is named for the cluster member closest to its centroid; we represent cluster names in bold, e.g., **123**, to distinguish them from individual maps or residues.

### Average map and molecule RMSD calculations

Average maps were calculated by Gaussian weighting (*w*) each map’s contribution based on its Euclidean distance from the cluster centroid:

$$w=\exp\left[–\left(d^{2}/\sigma^{2}\right)\right],$$

where *d* is the map’s distance from the centroid and *σ* = *d*_max_/8, the average of all maximum distances across all clusters in the chess square. Weighting was used so that maps closer to the cluster centroid contribute more to the average map. In contrast, taking an all-map flat average would overemphasize the importance of maps further from the centroid, of which there are more (Ahmed et al., 2015). We co-opted the term “exemplar” to represent the residue datum closest to the centroid of each cluster output by the k-means algorithm, which is slightly different from its formal definition in affinity propagation clustering.

RMSDs (root-mean square distances) for each residue type were calculated by first weighted-averaging all residue atomic positions in a cluster to construct an average residue structure. RMSDs were then calculated for both heavy atom and all-atom cases.

### Solvent-accessible and lipid-accessible surface area calculations

Solvent-accessible surface area (SASAs) for all residue sidechains were calculated using GETAREA (Fraczkiewicz and Braun, 1998) with default settings. The protein coordinates in PDB-formatted files were submitted as input. Water molecules are either explicit or presumed based on adequate available space. From GETAREA’s “In/Out” parameter, we created the “*f*_*outside*_” metric that represents the buriedness of residue collections, i.e., in a cluster, parse, chess square, *etc.*, By designating “In” as 0.0, “Out” as 1.0 and “indeterminant” as 0.5, and averaging these values for the collection. For residues in the “mL” data sets, e.g., ALAmL, the calculated SASAs are not wholly due to contact with water, either explicit or presumed, but often arise from potential contact with lipids; we thus term the resulting surface areas for these residues as LASAs or lipid-accessible surface areas. Operationally, if the ratio of the score sums involving lipid atoms to all atoms is greater than 0.1, that residue’s SASA is reclassified as a LASA.

## Results and discussion

### Datasets

All five of the residue types studied in this work are common in proteins. In our soluble dataset there are 57,104 alanines, 43,195 isoleucines, 69,012 leucines, 33,531 prolines and 53,826 valines. These account for 7.9%, 6.0%, 9.5%, 4.6% and 7.4% of all residues in this dataset, respectively. In the membrane dataset (RESm) there are 33,988 alanines, 27,434 isoleucines, 45,551 leucines, 16,111 prolines and 30,885 valines. These account for 8.8%, 7.1%, 11.7%, 4.1% and 8.0%, respectively of all membrane protein residues in our dataset. These residue types, except for PRO, are more prevalent in membrane proteins than soluble proteins.

**FIGURE 2:**
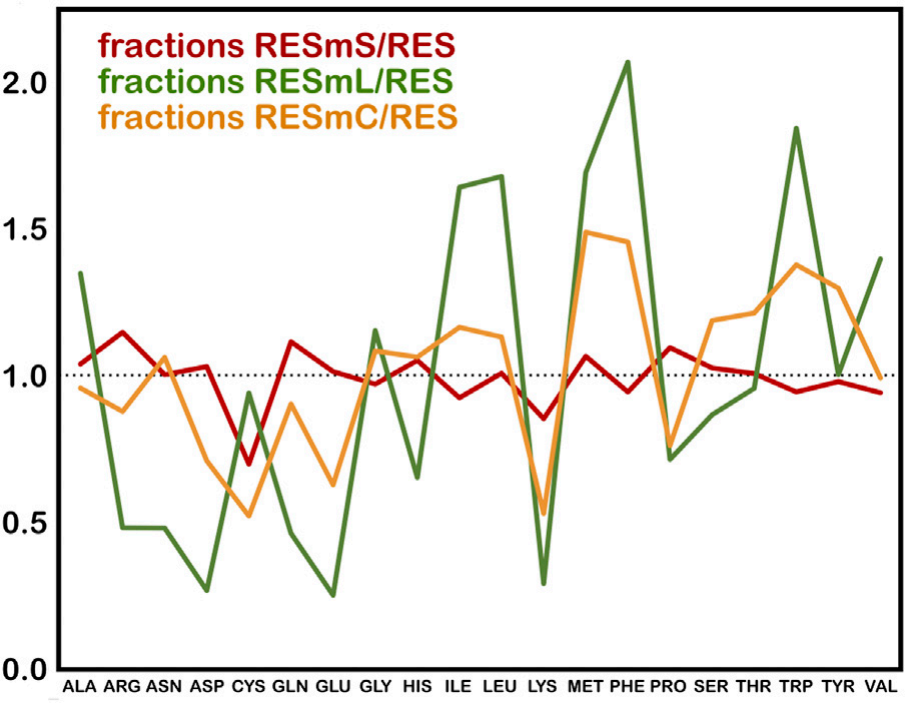
Fractions of membrane protein residues relative to residues in soluble proteins for membrane soluble domain (RESmS), lipid-facing (RESmL) and core (RESmC) transmembrane domains.

The three subsets we created from these data, i.e., “core” (RESmC), “lipid” (RESmL) and “soluble” (RESmS), show interesting trends - see Table 1. Not surprisingly, the RESmS data set appears from this perspective to be similar to previously reported residue frequencies for soluble proteins (AL Mughram et al., 2021b), at least for these residues. The frequencies for residues in the RESmL set, i.e., those more engaged with the lipids are higher than those seen in soluble proteins, except for proline, which is lower. The latter fact likely indicates that proline’s helix-breaking role is unwanted in this region. At first look, the core region data (RESmC) is very similar to the soluble region. We took a broader look, performing the same analysis for all residue types (see Figure 2).

**TABLE 1:** Frequency of ALA, ILE, LEU, PRO and VAL in membrane protein datasets.

	Soluble dataset^a^ (%)	RESmS/RESm^b^ (%)	RESmL/RESm^b^ (%)	RESmC/RESm^b^ (%)	RESmS/ALLmS^c^ (%)	RESmL/ALLmL^c^ (%)	RESmC/ALLmC^c^ (%)
ALA	7.6	48.7	45.5	5.7	7.9	10.3	7.3
ILE	5.8	41.0	52.4	6.6	5.4	9.5	6.8
LEU	9.2	42.7	51.2	6.1	9.3	15.5	10.4
PRO	4.5	64.1	30.2	5.7	4.9	3.2	3.4
VAL	7.1	45.4	48.5	6.1	6.7	10.0	7.1

^a^ AL Mughram et al., 2021b.;

^b^ Fraction RES in soluble domain, lipid-facing or core transmembrane domain of all RES in membrane proteins;

^c^ Fraction of RES in soluble domain, lipid-facing or core transmembrane domain of ALL residue types in these domains.

Clearly, most residue types are similarly represented in the soluble (RESmS) regions of membrane proteins as in soluble proteins. Cysteine shows the largest negative deviation, but since it is a fairly rare residue, it is difficult to assign much significance to this point. The second largest negative deviation is with lysine; interestingly it is sparsely found in all three regions despite its frequency of >6% in soluble proteins. The dramatic swings in the lipid accessible region (RESml) populations emphasize the structural character and role of transmembrane residues. The “core” region (RESmC) populations appear to be an amalgamation, and often an average, of the other two limiting case regions. However, further insight is to follow with other analyses we have performed in this work.

### Character and properties of residues

**FIGURE 3:**
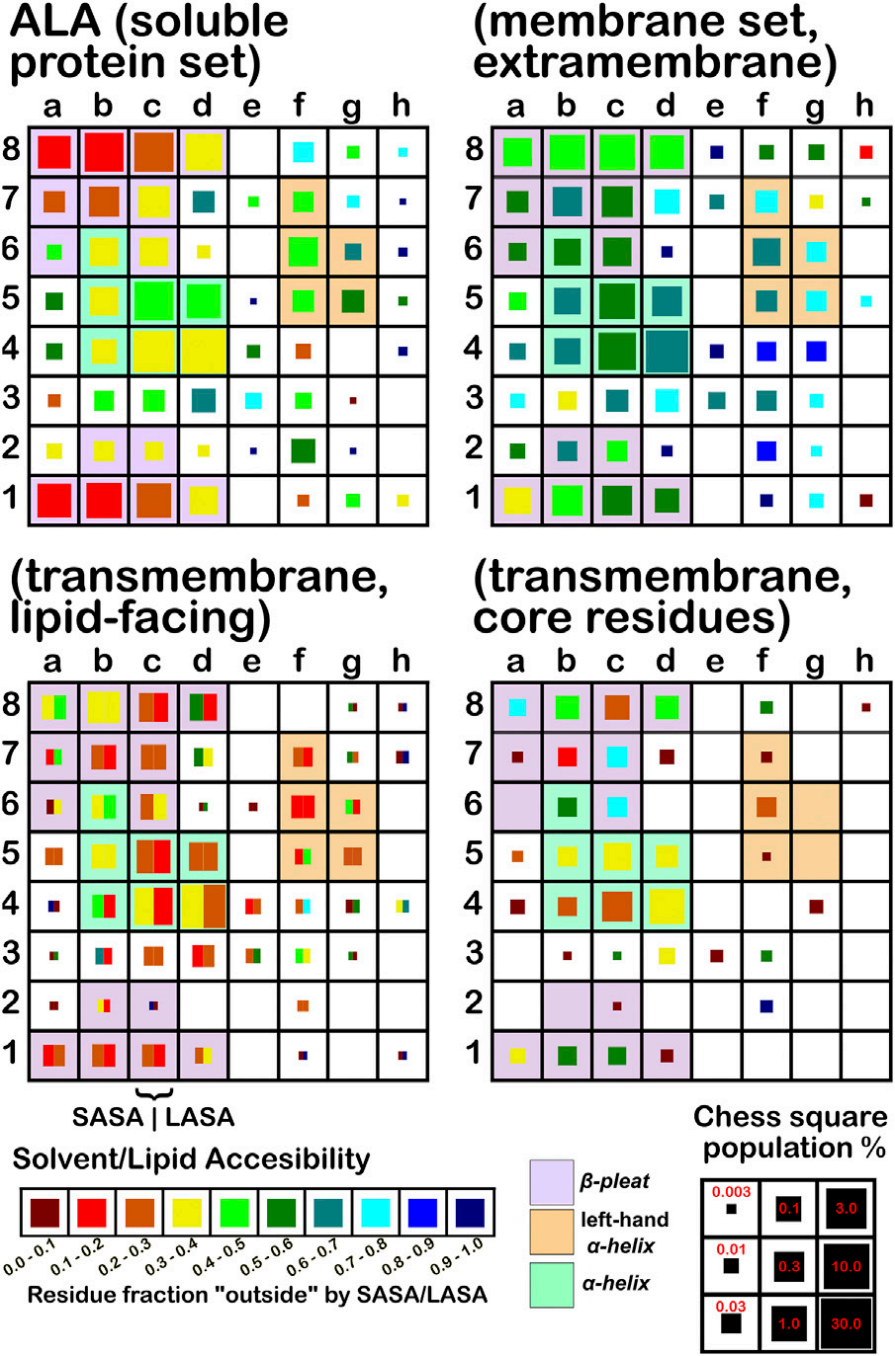
Population and solvent accessibility plots for alanine by chess square. Square sizes are logarithmically proportional to population, square colors encode the fraction of residues in that chess square exposed to solvent or lipid, as defined by the inset color map. Background colors show secondary structure.

**FIGURE 4:**
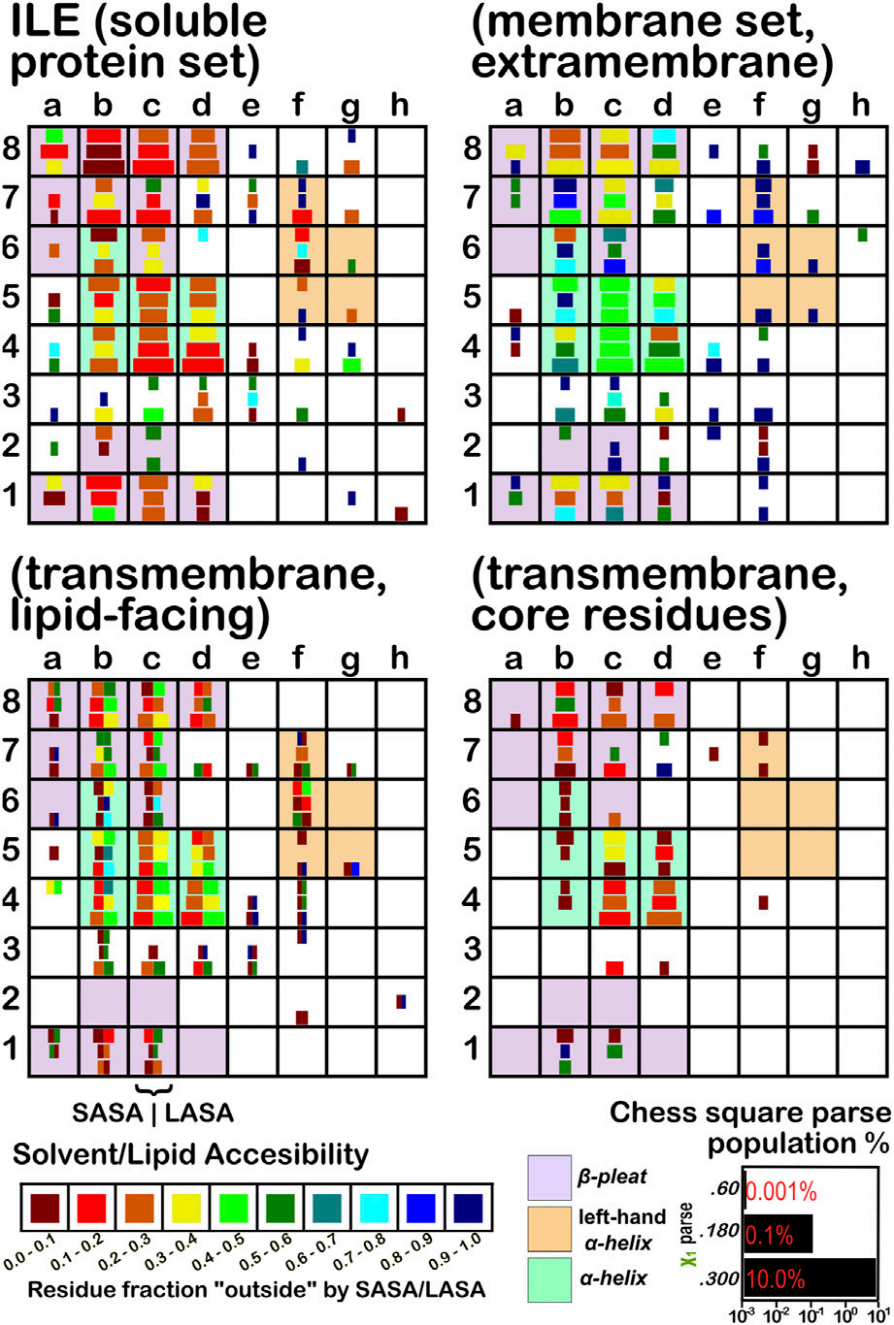
Population and solvent accessibility plots for isoleucine by chess square. Square sizes are logarithmically proportional to population, square colors encode the fraction of residues in that chess square exposed to solvent or lipid, as defined by the inset color map. Background colors show secondary structure.

**FIGURE 5:**
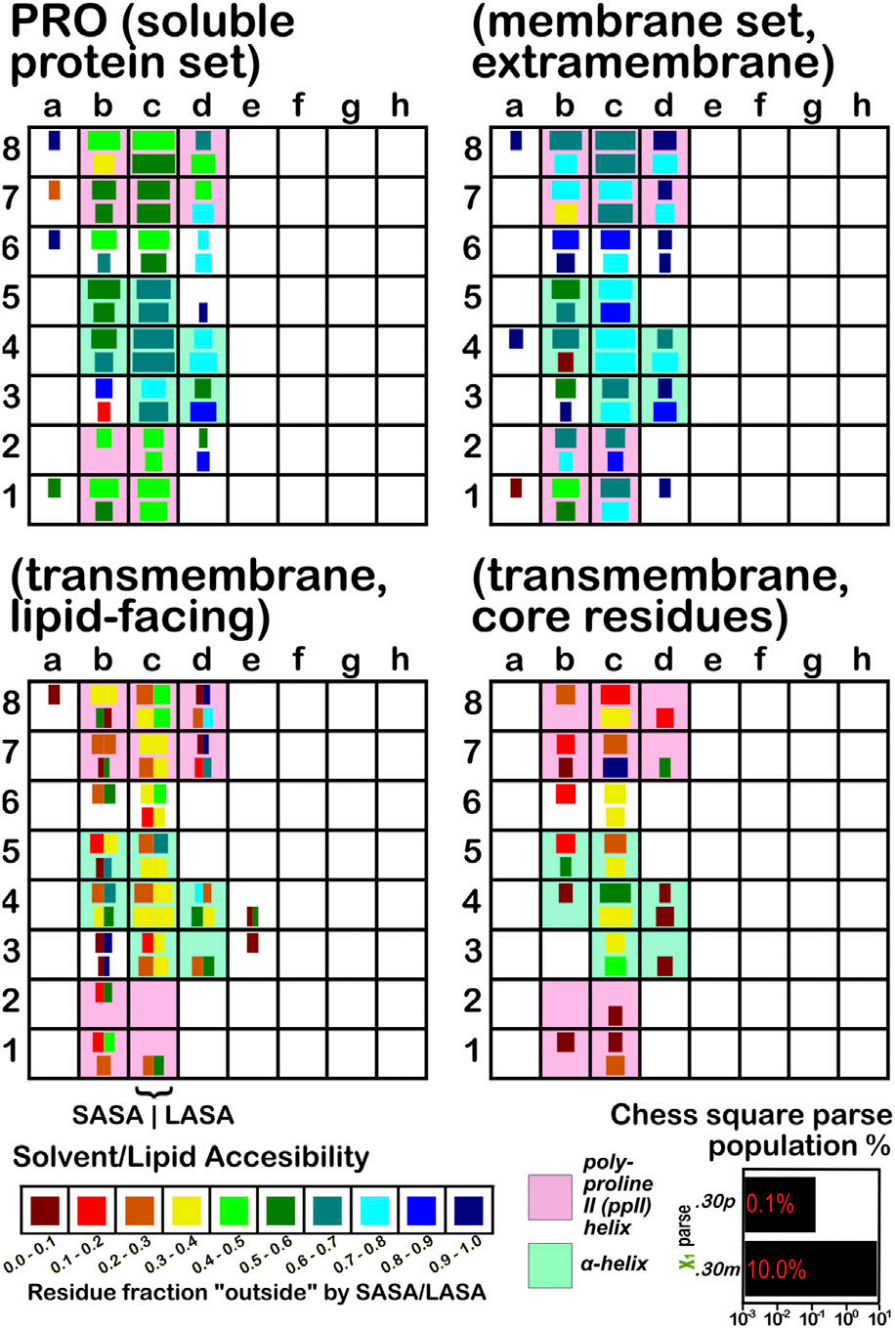
Population and solvent accessibility plots for proline by chess square. Bar lengths are logarithmically proportional to population, bar colors encode the fraction of residues in that chess square exposed to solvent or lipid, as defined by the inset color map. Background colors show secondary structure.

The soluble data set residue backbone angles follow very well the expectations from Ramachandran’s work (see Figure 3 for alanine; Figure 4 for isoleucine; Figure 5 for proline). Leucine (Supplementary Figure S1) and valine (Supplementary Figure S2) plots are in supporting information; the former is largely similar to alanine and the latter to isoleucine. For alanine and valine, the populations (log scale) are indicated by the size of the corresponding square in that chess square, while for isoleucine, leucine and proline, the χ_1_ parses are shown as horizontal bars (also in log scale). The extent to which a chess square is filled represents its relative population. Each of these squares are colored by their weighted solvent accessible surface area—here defined as the fraction of the residue “outside” or accessible. The hydrophobic residues in the β-pleat motif are somewhat more buried than those in the α-helix motif, an observation most evident for alanine.

The same analyses were performed for the three subsets of the membrane proteins and are also displayed in these figures. First, the RESmS subset data shows generally similar trends with respect to chess square (backbone angle) populations as seen in the soluble (RES) proteins, as should be expected. However, these residues are *significantly* more solvent exposed than their counterparts. As the protein fragments captured in this data set are lying just outside the membrane, they may indeed be more exposed; in this vein, such residues are known to often contain numerous less-structured loops and are thus likely less well-packed. It also may be an artifact of the isolation and crystallization techniques and protocols applied that may have stripped away interacting species. In that same vein, unnatural contacts, as a result of the forced crystallization of such artificial constructs may have a similar effect (Carpenter et al., 2008; Luo et al., 2015; Liu and Li, 2022). To our knowledge, detailed analyses of the hydropathic interactions at crystallographic contacts in membrane protein structures has never been performed, but we previously looked at these phenomena with respect to interfacial water in soluble proteins (Ahmed et al., 2013). Single-particle cryo-electron microscopy-solved structures would not close-pack extramembrane domains either, although a converse argument can be made that “drying” of crystals artificially close-packs such structures (Basak et al., 2018; Ravikumar et al., 2021). Also, protein-protein interactions that are experimentally-induced may be rarer in either case. Analyses of packing energetics showed very little difference between soluble and transmembrane proteins (Joh et al., 2009).

The second subset data, for the lipid exposed residues within the transmembrane region, shows a robustly enhanced proportion of residues in the α-helix motif: alanine—90.7%/62.4%; isoleucine—91.9%/43.5%; leucine—91.3%/57.0%; proline—38.9%/20.4%; and valine—91.4%/39.0%, for RES/RESmL α-helical fractions. Most extant crystal structures of membrane proteins have helix bundles in their transmembrane domains (As mentioned above, prolines are expected to be rare in this environment.). Both SASA (left half of square or bar) and LASA (right half) are shown. Note that the sum of SASA and LASA is the total fraction exposed. These data are laid out in detail in Supporting Information Supplementary Tables S6-S10. There are a few interesting observations: except for very sparsely populated chess squares, the SASA fraction is seldom zero. That is not to say that water was (or would be) found in these structures, but the possibility does exist. Water is known to associate with the lipid head groups and with attached methylenes (Disalvo and de los Angeles Frias, 2019).

Lastly, the third subset data, RESmC (residues on the interior of the transmembrane domain that are not directed towards the lipid bilayer) are plotted in the lower right data block. The populations are smaller and their, here water, solvent accessibility is modestly enhanced relative to the RESmL set, but still less than that of the (RES) soluble protein set. The residues in this subset are (or could be) in contact with water or other ions moving through the channel or transmembrane cavity they form. Thus, these residues could be functionally very significant. However, the small aliphatic hydrophobic residues of this study may also play the role of something akin to “Teflon coating” the channel walls. Note also that the membrane protein structures used here did not have water, ions or, *etc.*, In their models, which would be necessary for more detailed analyses.

In this article, we are taking particular interest in the ***c5*** chess square as it is representative of the α-helix motif. For alanine, we also examine the ***c5*** chess square in the β-pleat conformation. In ALA (Figure 3), the ***b1*** is robustly populated in soluble proteins and quite buried (10%–20% exposed); in the ALAmS, the relative population is largely consistent, but these residues are now 40%–50% exposed, suggesting fewer extended β-pleat subdomains in the extramembrane regions. Alanines in this conformation are fairly rare in the transmembrane region, but clearly those in contact with lipids (ALAmL) are buried, and thus not quite accessible. For alanine, the ***c5*** data shows that, in soluble proteins, residues in the α-helix conformation are common and 40%–50% exposed. Their exposure increases to 50%–60% in extramembrane regions. As noted above, alanines in the α-helix dominate the transmembrane region, and are similarly buried (sum of LASA and SASA, ∼30–40% for ALAmL).

Isoleucine (Figure 4), leucine (Supplementary Figure S1), proline (Figure 5) and valine (Supplementary Figure S2) are more hydrophobic than alanine, and are concomitantly more buried. Essentially, the same general trends are observed for isoleucine, leucine and valine structures, as for alanine, albeit interpretation is less transparent for the first two due to the χ_1_ parses. Proline (Figure 5) has different secondary structure definitions, and we are highlighting the ***c8*** chess square in this work. However, despite the low populations of transmembrane prolines, its residue accessibility trends are largely consistent with the other hydrophobic residues.

### Three-dimensional interaction maps

As described in the Methods, three dimensional maps cataloguing, for each residue in the study, the interactions between that residue and its environment were calculated. These maps illustrate the type (hydrophobic, hydrophobic-polar, favorable polar such as hydrogen bonding and acid-base, and unfavorable polar such as acid-acid and base-base), strength and loci of the interactions. As described in previous communications (Ahmed et al., 2015; Herrington and Kellogg, 2021), these maps, binned by chess square, and additionally in the cases of isoleucine, leucine and proline by χ_1_ angles, were clustered into map sets. Each cluster-derived map set is expected to represent a unique constellation of interactions between that residue and environment. We have termed these constellations the hydropathic valence of the residue type/secondary structure. *In toto*, these map sets are information-rich backbone-dependent rotamer and interaction libraries.

#### Alanine

**FIGURE 6:**
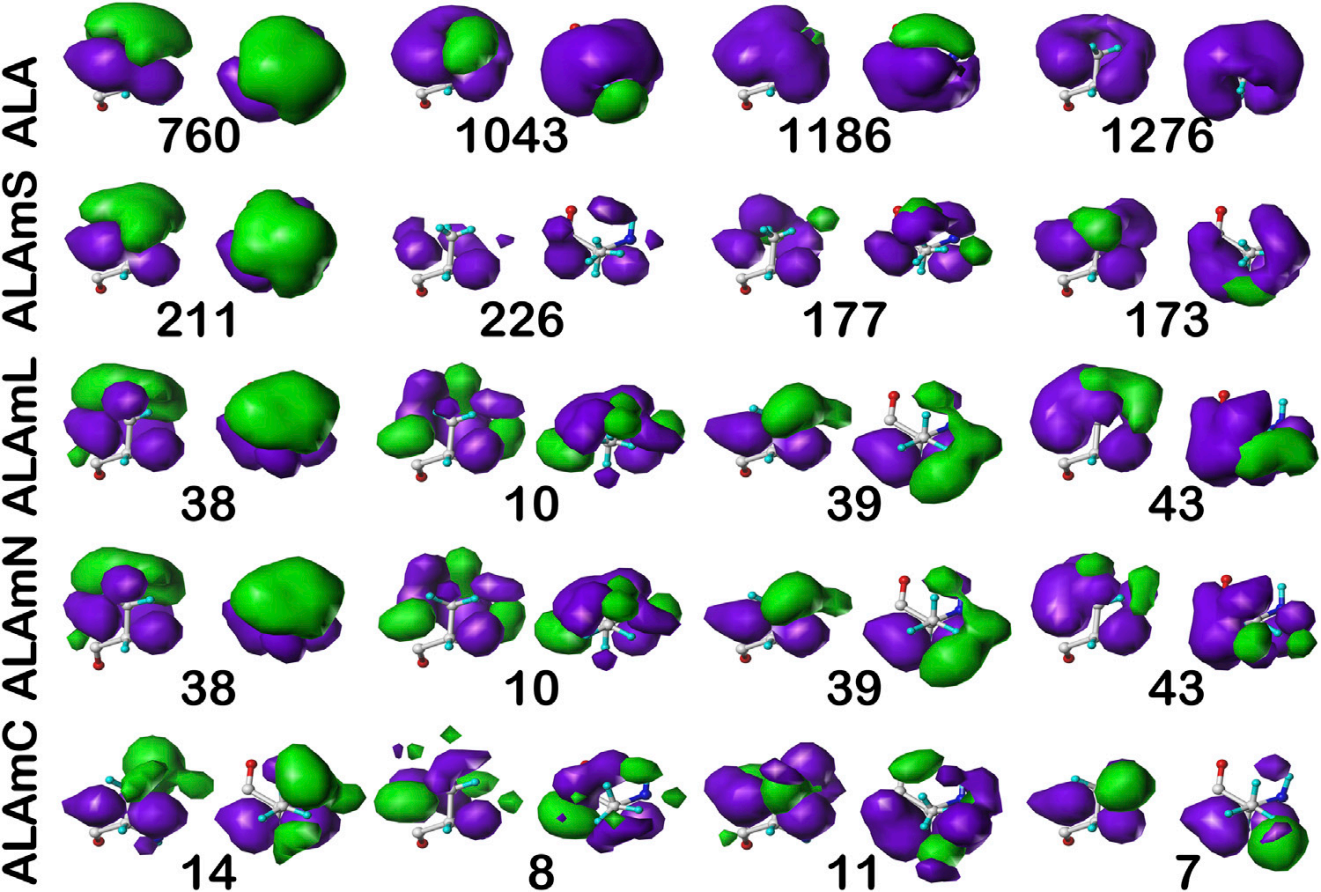
Three-dimensional clustered hydropathic interaction maps for alanine sidechains, in the ***b1*** chess square. Each map pair (or cluster) is named by its “exemplar”, which is the number of the map, as defined in the text, closest to the cluster’s centroid. Top row – alanine from soluble proteins dataset; 2^nd^ row – alanine from soluble domains of membrane proteins dataset; 3^rd^ row – lipid-facing alanines in transmembrane domains, including residue-lipid interactions; 4^th^ row – as 3^rd^ row, ignoring residue-lipid interactions; and 5^th^ row – core-facing alanines in transmembrane domains. Each residue/map is displayed in two orientations: left – z-axis (CA-CB bond) directed up, right – z-axis directed out of page. Green contours represent favorable hydrophobic interactions between the residue sidechain and its environment; purple contours represent unfavorable hydrophobic interactions.

Figure 6 illustrates the interaction map sets for the four clusters found for the sidechain interaction maps of alanine in the ***b1*** chess square. Table 2 lists a number of metrics describing these clusters, including their relative populations, solvent-accessible surface areas (SASAs), and similarity metrics. These three data assist in characterizing the weighted average 3D maps calculated from members of each cluster. First, the relative population of each cluster indicates the fraction of residues within a chess square or chess square/parse that display the 3D interaction preferences or motif illustrated by the associated map. As will be discussed below, these relative fractions carry significant information alone. Second, the average SASAs (LASAs) indicate the average solvent (lipid) exposure for the residue sidechains of the cluster members. We include these data not only because they are informative and characteristic of the average residue in the cluster, but also because they are calculated completely independently from our HINT and mapping protocol. Third, the map-map similarity or correlation data, calculated as described above, indicates the sameness of two weighted average cluster maps; here we are using it to compare maps from the different residue datasets in this study. A similarity of 1.0000 suggests that two maps are nearly or precisely identical, while lesser values represent divergences. Supporting information, Supplementary Tables S6A-E, contains these numerical data for all alanine chess squares. In the discussion that follows, contoured maps will be presented, deciphered and compared, with the above-described numerical data as context.

**TABLE 2:** Cluster parameters and cluster-cluster similarities for alanine data sets.

*Dataset*	***Chess square*:cluster**	Relative fraction^a^ (%)	SASA (Å^2^)^b^	LASA (Å^2^)^c^	Most similar ALA^d^		Most similar ALAmS^e^		Most similar ALAmL^f^		Most similar ALAmN^g^		Most similar ALAmC^h^	
					cluster	metric	cluster	metric	cluster	metric	cluster	metric	cluster	metric
ALA	***b1***:**760**	62.3	1±3	--	--	--	***b1***:**211**	0.9974	***b1***:**38**	0.9749	***b1***:**38**	0.9749	***b1***:**14**	0.9362
	***b1***:**1043**	8.3	43±16	--	--	--	***b1***:**226**	0.9593	***b1***:**10**	0.8709	***b1***:**43**	0.8851	***b1***:**8**	0.8770
	***b1***:**1186**	14.9	17±12	--	--	--	***b1***:**177**	0.9726	***b1***:**38**	0.9615	***b1***:**38**	0.9615	***b1***:**14**	0.9179
	***b1***:**1276**	14.5	9±11	--	--	--	***b1***:**173**	0.9827	***b1***:**43**	0.9544	***b1***:**39**	0.9469	***b1***:**7**	0.9433
	***c5***:**829**	26.8	42±14	--	--	--	***c5***:**128**	0.9837	***c5***:**518**	0.9336	***c5***:**518**	0.9333	***c5***:**86**	0.9352
	***c5***:**1830**	12.5	61±12	--	--	--	***c5***:**771**	0.9599	***c5***:**518**	0.8858	***c5***:**518**	0.9092	***c5***:**86**	0.9031
	***c5***:**3020**	25.2	8±11	--	--	--	***c5***:**905**	0.9885	***c5***:**393**	0.9952	***c5***:**18**	0.9890	***c5***:**7**	0.9859
	***c5***:**3449**	35.5	11±11	--	--	--	***c5***:**905**	0.9712	***c5***:**679**	0.9957	***c5***:**679**	0.9943	***c5***:**139**	0.9768
ALAmS	***b1***:**173**	21.3	14±17	--	***b1***:**1276**	0.9827	--	--	***b1***:**43**	0.9433	***b1***:**43**	0.9426	***b1***:**11**	0.9345
	***b1***:**177**	22.4	28±21	--	***b1***:**1186**	0.9726	--	--	***b1***:**38**	0.9258	***b1***:**38**	0.9258	***b1***:**11**	0.9057
	***b1***:**211**	40.5	2±5	--	***b1***:**760**	0.9974	--	--	***b1***:**38**	0.9726	***b1***:**38**	0.9726	***b1***:**14**	0.9365
	***b1***:**226**	15.8	56±32	--	***b1***:**1043**	0.9593	--	--	***b1***:**10**	0.8620	***b1***:**43**	0.8673	***b1***:**8**	0.8696
	***c5***:**128**	22.5	47±25	--	***c5***:**829**	0.9837	--	--	***c5***:**518**	0.9490	***c5***:**518**	0.9379	***c5***:**86**	0.9460
	***c5***:**771**	14.0	79±26	--	***c5***:**1830**	0.9599	--	--	***c5***:**518**	0.8891	***c5***:**518**	0.9159	***c5***:**86**	0.9207
	***c5***:**905**	45.3	8±13	--	***c5***:**3020**	0.9885	--	--	***c5***:**18**	0.9970	***c5***:**18**	0.9979	***c5***:**7**	0.9905
	***c5***:**996**	18.2	22±25	--	***c5***:**3020**	0.9786	--	--	***c5***:**393**	0.9819	***c5***:**393**	0.9836	***c5***:**7**	0.9634
ALAmL	***b1***:**10**	15.9	25±22	16±36	***b1***:**1186**	0.9242	***b1***:**177**	0.9177	--	--	***b1***:**10**	0.9995	***b1***:**8**	0.9319
	***b1***:**38**	29.5	5±10	3±9	***b1***:**760**	0.9749	***b1***:**211**	0.9726	--	--	***b1***:**38**	1.0000	***b1***:**14**	0.9163
	***b1***:**39**	31.8	11±16	7±19	***b1***:**1276**	0.9526	***b1***:**211**	0.9502	--	--	***b1***:**39**	0.9947	***b1***:**14**	0.9548
	***b1***:**43**	22.7	3±4	12±27	***b1***:**1276**	0.9544	***b1***:**173**	0.9433	--	--	***b1***:**43**	0.9782	***b1***:**11**	0.8895
	***c5***:**18**	43.9	1±4	10±22	***c5***:**3020**	0.9881	***c5***:**905**	0.9970	--	--	***c5***:**18**	0.9990	***c5***:**7**	0.9926
	***c5***:**393**	24.6	5±10	17±29	***c5***:**3020**	0.9952	***c5***:**996**	0.9819	--	--	***c5***:**393**	0.9956	***c5***:**7**	0.9776
	***c5***:**518**	8.9	27±30	20±33	***c5***:**829**	0.9336	***c5***:**128**	0.9490	--	--	***c5***:**518**	0.9793	***c5***:**57**	0.9311
	***c5***:**679**	22.6	6±11	11±23	***c5***:**3449**	0.9957	***c5***:**905**	0.9875	--	--	***c5***:**679**	0.9991	***c5***:**139**	0.9855
ALAmC	***b1***:**7**	33.3	56±10	--	***b1***:**1276**	0.9433	***b1***:**173**	0.9234	***b1***:**39**	0.9361	***b1***:**39**	0.9387	--	--
	***b1***:**8**	13.3	30±26	--	***b1***:**1186**	0.9025	***b1***:**173**	0.8962	***b1***:**10**	0.9319	***b1***:**10**	0.9321	--	--
	***b1***:**11**	26.7	12±1	--	***b1***:**1276**	0.9376	***b1***:**173**	0.9345	***b1***:**39**	0.9239	***b1***:**39**	0.9255	--	--
	***b1***:**14**	26.7	4±4	--	***b1***:**760**	0.9362	***b1***:**211**	0.9365	***b1***:**39**	0.9548	***b1***:**39**	0.9506	--	--
	***c5***:**7**	43.9	2±4	--	***c5***:**3020**	0.9859	***c5***:**905**	0.9905	***c5***:**18**	0.9926	***c5***:**18**	0.9913	--	--
	***c5***:**57**	20.4	18±17	--	***c5***:**3020**	0.9615	***c5***:**996**	0.9482	***c5***:**393**	0.9672	***c5***:**393**	0.9633	--	--
	***c5***:**86**	8.3	46±21	--	***c5***:**829**	0.9352	***c5***:**128**	0.9460	***c5***:**518**	0.9178	***c5***:**518**	0.9262	--	--
	***c5***:**139**	27.4	20±16	--	***c5***:**3449**	0.9768	***c5***:**905**	0.9586	***c5***:**679**	0.9855	***c5***:**679**	0.9849	--	--

^a^ Fraction of residues in cluster relative to all in *chess square.parse*;

^b^ From GETAREA (Fraczkiewicz and Braun, 1998);

^c^ Adapted from GETAREA results as described in text;

^d^ Cluster map in ALA dataset most similar to cluster map named by row. Note that this may not be commutative;

^e^ Cluster map in ALAmS dataset most similar to cluster map named by row;

^f^ Cluster map in ALAmL dataset most similar to cluster map named by row;

^g^ Cluster map in ALAmN dataset most similar to cluster map named by row;

^h^ Cluster map in ALAmC dataset most similar to cluster map named by row.

Each map is depicted with two views: on the left, the *z*-axis (CA-CB bond) is pointed up, while on the right, the *z*-axis is pointed out of the paper’s plane. This convention is used for all map views in this article. The top row maps are for alanines in the soluble protein data set and were previously reported in another article (Ahmed et al., 2019). The most common map, ***b1*:760**, accounts for 62.4% of the alanines in this conformation and presents with strong hydrophobic interactions in the *z* direction with a collar of hydrophobic-polar interactions. Also of note, ***b1*:760** has a very low SASA (1±3) indicating that this particular 3D interaction profile is almost exclusively buried. In contrast, ***b1*:1043** is mostly solvent-exposed (43±16), but relatively rare—clustered at only 8.3% of ***b1*** alanines. The second row of maps (Figure 6) are those extracted from the soluble domains of the membrane proteins, i.e., the ALAmS dataset. They are ordered by similarity to alanines in the ALA dataset. Thus, ***b1*:211** of ALAmS is most similar to ***b1*:760** of ALA. Indeed, Table 2 indicates that this pair of maps has a similarity metric of 0.9974, and it is plain that they are visually nearly identical. (All similarity metrics for alanine’s ***b1*** and ***c5*** chess squares are available in supporting information, Supplementary Table S11) While ***b1*:211** of ALAmS is the most common map, it is found at 40.5%, and the other three ALAmS maps contribute more overall than in ALA. On the surface, ALA ***b1*:1043** and ALAmS ***b1*:226** do not seem similar, and their similarity is only 0.9593, but their SASA values are consistent. It should be noted that our mapping algorithm does calculate interactions for crystallographic water molecules in the structures, but the Fraczkiewicz and Braun (1998) GETAREA algorithm strips explicit water molecules in its calculations. Note also that there are no explicit (crystallographic or otherwise modeled) water molecules in the membrane protein data set. Thus, the interaction profiles represented by ALA ***b1*:1043** and ALAmS ***b1*:226** are likely much more similar than they appear.

The lipid facing dataset for alanine, ALAmL, was evaluated in two ways: 1) interactions involving the artificial/modeled lipids were included in the map calculations, as shown on the third row of Figure 6; and 2) these interactions were ignored, as is shown on the fourth (ALAmN) row. Clustering was performed on the ALAmL set and that clustering solution was applied to the ALAmN set. (The ALAmN dataset can also be independently clustered: it is generally similar to the clustering afforded by ALAmL, but the advantage of direct comparisons is evident.). We see the ALAmL maps as *training* membrane-contacting residue clusters for the types of interactions that may be expected. Also, the extent of lipid-residue interactions was used to define the difference between solvent-accessible and lipid-accessible surface areas. Table 2 lays out the data for these clusters. There is obviously, in this case, very little difference between the ALAmL and ALAmN data sets—the similarities between cluster pairs are 0.9782 and better. This is likely because accessibility is low in the ***b1*** conformation. The only evident difference is in ***b1*:43**, where some *z*-axis hydrophobic interactions present in ALAmL were lost in the ALAmN maps. It should also be recalled that the ***b1*** conformation, as are all β-pleat chess squares, weakly populated—with ∼1% of the population in soluble ALA and ∼10% of the ALAmS population—so the resultant data ***b1*** data in transmembrane regions is less statistically certain. This latter point is even more true for ALAmC, whose maps are displayed on the fifth row of Figure 6. The observation made above, with respect to the reduced solvent-accessibility of the core residues (Figures 3, 4, 5, Supplementary Figure S1, Supplementary Figure S2) compared to the soluble protein, are evident here as well: there are significantly fewer unfavorable hydrophobic interactions in the ALAmC set.

**FIGURE 7:**
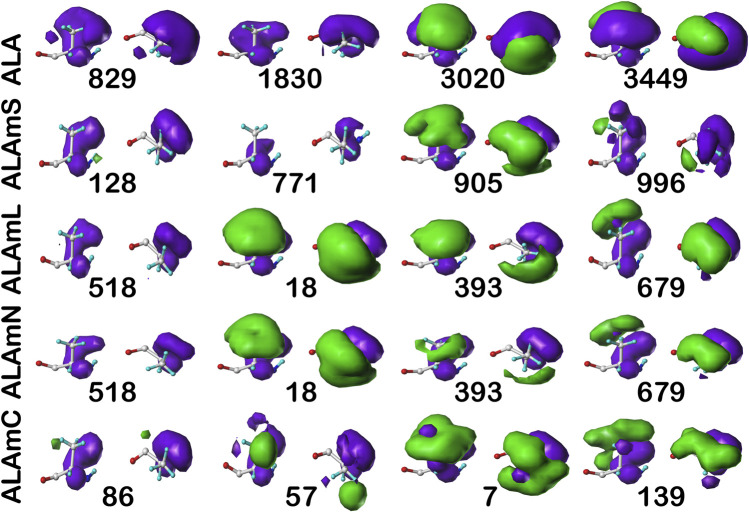
Three-dimensional clustered hydropathic interaction maps for alanine sidechains, in the ***c5*** chess square. See caption for Figure 6.

For comparison, the cluster maps for the ***c5*** chess square conformation are shown in Figure 7, with the associated data again in Table 2. This conformation more often exposes alanines to solvent with close to 40% of alanines (clusters **829** and **1830**) in the soluble data set having SASAs greater than 40 Å^2^ vs only 8% in ***b1*** (**1043**). In the similarities for ***c5***, we see what may be described as confusion with respect to pair matching; e.g., cluster **905** of ALAmS shows high similarity to both **3020** (0.9885) and **3449** (0.9712) of ALA. The **3020**–**3449** map pair itself has a fairly high similarity of 0.9418, which suggests that perhaps three rather than four clusters might have been appropriate. However, calculating cluster sets with inconsistent numbers of clusters tends to obscure both visual and numerical similarity comparisons.

It is clearer that the ALAmN set is different than the ALAmL set in this chess square compared to ***b1*** because alanines in this conformation are more solvent exposed. All cluster pairs show visual differences, but cluster **393** is perhaps the most revealing. It interacts significantly with lipids in the former, but is more apparently exposed in the latter. Ignoring lipid interactions, as in ALAmN, it is, overall, exposed about 22 Å^2^, with a SASA of ∼5 Å^2^ complemented by a LASA of ∼17 Å^2^. We believe that this information, which is calculated for every residue in this study, for all clusters and chess squares, is novel and useful.

#### Valine

**FIGURE 8:**
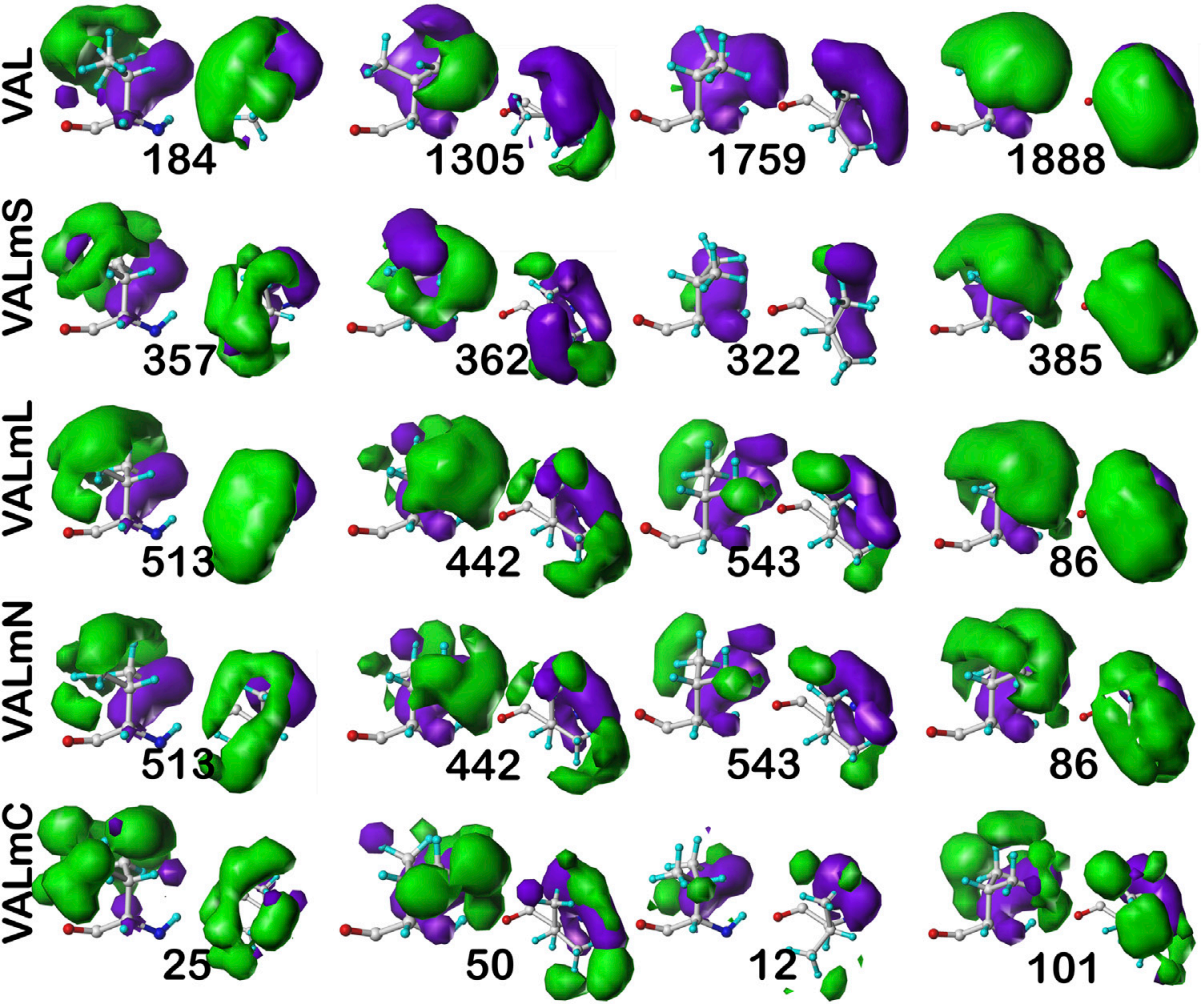
Three-dimensional clustered hydropathic interaction maps for valine sidechains, in the ***c5*** chess square. Each map pair (or cluster) is named by its “exemplar”, which is the number of the map, as defined in the text, closest to the cluster’s centroid. Top row – valine from soluble proteins dataset; 2^nd^ row – valine from soluble domains of membrane proteins dataset; 3^rd^ row – lipid-facing valines in transmembrane domains, including residue-lipid interactions; 4^th^ row – as 3^rd^ row, ignoring residue-lipid interactions; and 5^th^ row – core-facing valines in transmembrane domains. See also caption for Figure 6.

As we have indicated, the population of chess squares comprising the β-pleat secondary structure, i.e., the ***b1*** chess square, are weakly populated and are not discussed. The maps shown in Figure 8 are for four selected clusters from the ***c5*** conformation. The numerical data for the ***c5*** chess square is set out in Table 3 (and more detailed data is in supporting information Supplementary Tables S7A-E for all valines, and all similarity metrics for its ***b1*** and ***c5*** chess squares are in Supplementary Table S12. Valine has three hydrophobic atoms compared to alanine’s one, and is obviously more engaged in hydrophobic interactions. The VAL/VALmS paired maps are quite similar in appearance and only the solvent-accessible **1759/322** pair has a similarity less than 0.97. It is perhaps unexpected, but the actual average SASA (5 Å^2^) for all valines in the lipid-facing region (VALmL) is only slightly larger than that for all alanines (4 Å^2^); however, its LASA is larger (23 Å^2^) vs alanine (16 Å^2^). In soluble proteins the SASAs for ALA and VAL are 17 Å^2^ and 18 Å^2^, respectively. None of these comparisons are statistically significant, but we feel that they do indicate shifting of roles for the two residues in different environments. Evidence for the importance of lipid-residue interactions is somewhat more easily found here than in alanine; e.g., the **86**, **442** and **513** maps show diminished hydrophobic interactions in VALmN vs VALmL. Generally, clusters with low SASA and relatively high LASA show this effect, e.g., **513**, where SASA ∼3 Å^2^ and LASA ∼20 Å^2^.

**TABLE 3:** Cluster parameters and cluster-cluster similarities for valine data sets.

	***Chess square*:cluster**	Relative fraction^a^ (%)	SASA (Å^2^)^b^	LASA (Å^2^)^c^	Most similar VAL^d^		Most similar VALmS^e^		Most similar VALmL^f^		Most similar VALmN^g^		Most similar VALmC^h^	
					cluster	metric	cluster	metric	cluster	metric	cluster	metric	cluster	metric
VAL	***c5*:184**	12.3	14±15	--	--	--	***c5*:357**	0.9865	***c5*:513**	0.9897	***c5*:513**	0.9862	***c5*:25**	0.9647
	***c5*:777**	7.9	7±8	--	--	--	***c5*:468**	0.9926	***c5*:60**	0.9917	***c5*:60**	0.9868	***c5*:44**	0.9769
	***c5*:883**	8.3	27±16	--	--	--	***c5*:357**	0.9514	***c5*:187**	0.9695	***c5*:187**	0.9590	***c5*:44**	0.9230
	***c5*:1305**	9.6	31±16	--	--	--	***c5*:362**	0.9763	***c5*:442**	0.9769	***c5*:442**	0.9718	***c5*:50**	0.9713
	***c5*:1350**	16.4	14±11	--	--	--	***c5*:385**	0.9821	***c5*:369**	0.9902	***c5*:369**	0.9830	***c5*:101**	0.9616
	***c5*:1702**	5.1	101±13	--	--	--	***c5*:322**	0.9161	***c5*:187**	0.8443	***c5*:187**	0.8549	***c5*:12**	0.8644
	***c5*:1759**	8.3	81±14	--	--	--	***c5*:322**	0.9264	***c5*:543**	0.8844	***c5*:543**	0.8896	***c5*:12**	0.8803
	***c5*:1857**	10.0	40±15	--	--	--	***c5*:124**	0.9834	***c5*:369**	0.9785	***c5*:369**	0.9773	***c5*:101**	0.9470
	***c5*:1888**	22.2	3±5	--	--	--	***c5*:385**	0.9972	***c5*:86**	0.9973	***c5*:86**	0.9959	***c5*:101**	0.9797
VALmS	***c5*:40**	0.7	92±14	--	***c5*:1702**	0.8669	--	--	***c5*:436**	0.8167	***c5*:436**	0.8588	***c5*:12**	0.7945
	***c5*:124**	15.2	28±21	--	***c5*:777**	0.9834	--	--	***c5*:369**	0.9828	***c5*:369**	0.9809	***c5*:101**	0.9598
	***c5*:195**	7.8	34±25	--	***c5*:1305**	0.8983	--	--	***c5*:60**	0.9137	***c5*:60**	0.9157	***c5*:24**	0.9083
	***c5*:262**	8.2	24±18	--	***c5*:883**	0.9512	--	--	***c5*:60**	0.9510	***c5*:60**	0.9422	***c5*:44**	0.9101
	***c5*:322**	4.8	71±25	--	***c5*:1759**	0.9264	--	--	***c5*:187**	0.8561	***c5*:187**	0.8638	***c5*:12**	0.8683
	***c5*:357**	12.3	8±10	--	***c5*:184**	0.9865	--	--	***c5*:513**	0.9922	***c5*:513**	0.9900	***c5*:25**	0.9451
	***c5*:362**	12.6	11±15	--	***c5*:1888**	0.9780	--	--	***c5*:442**	0.9767	***c5*:442**	0.9726	***c5*:101**	0.9612
	***c5*:385**	23.4	3±11	--	***c5*:1888**	0.9972	--	--	***c5*:86**	0.9959	***c5*:86**	0.9943	***c5*:101**	0.9786
	***c5*:468**	15.0	5±11	--	***c5*:777**	0.9926	--	--	***c5*:60**	0.9927	***c5*:60**	0.9835	***c5*:44**	0.9764
VALmL	***c5*:48**	6.9	4±6	16±27	***c5*:1888**	0.9855	***c5*:385**	0.9799	--	--	***c5*:48**	0.9969	***c5*:103**	0.9629
	***c5*:60**	25.0	2±5	25±30	***c5*:777**	0.9916	***c5*:468**	0.9927	--	--	***c5*:60**	0.9971	***c5*:44**	0.9754
	***c5*:86**	17.2	1±2	19±24	***c5*:1888**	0.9973	***c5*:385**	0.9959	--	--	***c5*:86**	0.9983	***c5*:101**	0.9813
	***c5*:187**	7.1	6±12	30±34	***c5*:184**	0.9802	***c5*:357**	0.9842	--	--	***c5*:187**	0.9980	***c5*:92**	0.9503
	***c5*:369**	10.5	7±11	16±25	***c5*:1350**	0.9902	***c5*:385**	0.9885	--	--	***c5*:369**	0.9989	***c5*:101**	0.9770
	***c5*:436**	4.2	20±30	48±39	***c5*:883**	0.8740	***c5*:468**	0.8725	--	--	***c5*:436**	0.9214	***c5*:50**	0.8728
	***c5*:442**	7.8	7±9	11±21	***c5*:1888**	0.9839	***c5*:362**	0.9767	--	--	***c5*:442**	0.9994	***c5*:101**	0.9751
	***c5*:513**	16.4	3±6	20±27	***c5*:184**	0.9897	***c5*:357**	0.9922	--	--	***c5*:513**	0.9986	***c5*:92**	0.9669
	***c5*:543**	5.1	17±19	24±30	***c5*:1857**	0.9546	***c5*:362**	0.9527	--	--	***c5*:543**	0.9958	***c5*:101**	0.9440
VALmC	***c5*:12**	14.2	22±14	--	***c5*:1857**	0.9151	***c5*:124**	0.9106	***c5*:543**	0.9219	***c5*:543**	0.9188	--	--
	***c5*:24**	9.4	17±9	--	***c5*:1888**	0.9281	***c5*:468**	0.9407	***c5*:48**	0.9322	***c5*:48**	0.9289	--	--
	***c5*:25**	7.5	11±7	--	***c5*:184**	0.9647	***c5*:357**	0.9451	***c5*:513**	0.9501	***c5*:513**	0.9443	--	--
	***c5*:44**	19.8	4±6	--	***c5*:777**	0.9768	***c5*:468**	0.9764	***c5*:60**	0.9754	***c5*:60**	0.9611	--	--
	***c5*:48**	3.8	27±10	--	***c5*:1857**	0.9328	***c5*:124**	0.9358	***c5*:369**	0.9418	***c5*:369**	0.9327	--	--
	***c5*:50**	10.4	17±13	--	***c5*:1305**	0.9712	***c5*:362**	0.9429	***c5*:442**	0.9673	***c5*:442**	0.9654	--	--
	***c5*:92**	9.4	4±5	--	***c5*:184**	0.9572	***c5*:357**	0.9613	***c5*:513**	0.9669	***c5*:513**	0.9574	--	--
	***c5*:101**	16.0	6±8	--	***c5*:1888**	0.9797	***c5*:385**	0.9786	***c5*:86**	0.9812	***c5*:86**	0.9802	--	--
	***c5*:103**	9.4	10±8	--	***c5*:1888**	0.9534	***c5*:385**	0.9411	***c5*:48**	0.9629	***c5*:48**	0.9489	--	--

^a^ Fraction of residues in cluster relative to all in *chess square.parse*;

^b^ From GETAREA (Fraczkiewicz and Braun, 1998);

^c^ Adapted from GETAREA results as described in text;

^d^ Cluster map in VAL dataset most similar to cluster map named by row. Note that this may not be commutative;

^e^ Cluster map in VALmS dataset most similar to cluster map named by row;

^f^ Cluster map in VALmL dataset most similar to cluster map named by row;

^g^ Cluster map in VALmN dataset most similar to cluster map named by row;

^h^ Cluster map in VALmC dataset most similar to cluster map named by row.

#### Isoleucine

**FIGURE 9:**
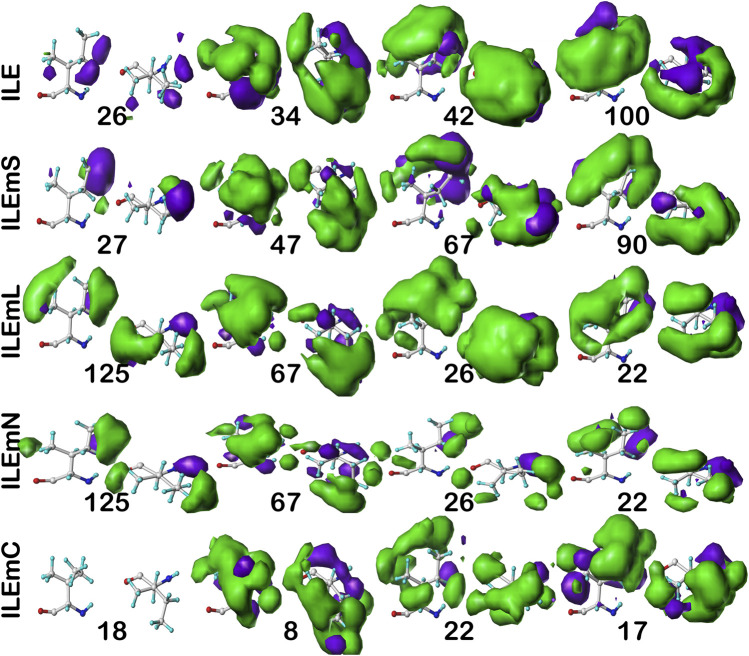
Three-dimensional clustered hydropathic interaction maps for isoleucine sidechains, in the ***c5*** chess square, χ_1_ = 300°. Top row – isoleucines from soluble proteins dataset; 2^nd^ row – isoleucines from soluble domains of membrane proteins dataset; 3^rd^ row – lipid-facing isoleucines in transmembrane domains, including residue-lipid interactions; 4^th^ row – as 3^rd^ row, ignoring residue-lipid interactions; and 5^th^ row – core-facing isoleucines in transmembrane domains. See also caption for Figure 6.

For isoleucine (and leucine) there are three χ_1_ “parses” per chess square with a similarly increased number of clusters. Thus, we have prepared visual cluster maps displays (Figure 9) for only one parse (***c5*.300**) and only four of its nine clusters. Because of isoleucine’s particular conformation, the *χ*_1_ = 300° parse is not as highly populated as either the 60° or 180° parses, and this chess square is significantly less populated than ***c5*** of leucine. The maps are organized, as above for alanine and valine, by similarity to the soluble (ILE) dataset cluster maps. With four, compared to one, hydrophobic sidechain atom, isoleucine maps are much more hydrophobic than alanine maps. Also, the atom-atom interaction matrices from which the maps are calculated are up to four times as complex, so the maps are also more complex. Nevertheless, there are clearly commonalities in map profiles and features. The metrics describing the clustered maps for ***c5*.300** are listed in Table 4. Many map pairs have similarities of ∼0.96 or larger, especially for ILE/ILEmS, e.g., ILE **34** and ILEmS **47**, which again shows that these two sets are quite similar. There are now very obvious differences between the ILEmL and ILEmN cluster maps (Figure 9), with reduced cluster-cluster similarity metrics in Table 4 (See supporting information Supplementary Tables S8A-E for all isoleucine data, and similarity metrics for alanine’s ***b1*** and ***c5*** chess squares are available in supporting information, Supplementary Table S13) For instance, cluster **26**—that represents more than an eighth of the map profiles—is markedly different between the two (similarity = 0.9301 and ILEmN cluster **17** is actually numerically more similar to ILEmL **26**). The large cluster **26** LASA of ∼41 Å^2^ and no SASA indicates its structural role of interacting with lipids within the transmembrane region. Cluster **22** shows less difference between ILEmL and ILEmN (0.9907) and has about half the LASA of **26**. Its structural role would appear to be more integral to supporting its associated helix. The core transmembrane isoleucine (ILEmC) cluster maps are noticeably less similar to the soluble (ILE) set. Their similarities (Table 4) are now closer to 0.9, with the highest (0.9343) between ILE **34** and ILEmC **8**. It is important to reiterate, however, that the RESmC data sets are not highly populated, so both visual and numerical comparisons may be less reliable here.

**TABLE 4:** Cluster parameters and cluster-cluster similarities for isoleucine data sets.

	***Chess square.parse*:cluster**	Relative fraction^a^ (%)	SASA (Å^2^)^b^	LASA (Å^2^)^c^	Most similar ILE^d^		Most similar ILEmS^e^		Most similar ILEmL^f^		Most similar ILEmN^g^		Most similar ILEmC^h^	
					cluster	metric	cluster	metric	cluster	metric	cluster	metric	cluster	metric
ILE	***c5.300***:**19**	9.6	40±24	--	--	--	***c5***:**90**	0.9087	***c5***:**125**	0.9130	***c5***:**125**	0.9461	***c5***:**17**	0.9019
	***c5.300***:**26**	10.1	98±18	--	--	--	***c5***:**27**	0.8737	***c5***:**125**	0.8465	***c5***:**13**	0.8554	***c5***:**18**	0.8581
	***c5.300***:**34**	20.2	7±10	--	--	--	***c5***:**47**	0.9625	***c5***:**67**	0.9593	***c5***:**67**	0.9484	***c5***:**8**	0.9343
	***c5.300***:**38**	5.6	38±19	--	--	--	***c5***:**67**	0.9569	***c5***:**17**	0.9368	***c5***:**22**	0.9437	***c5***:**22**	0.9120
	***c5.300***:**42**	19.7	3±4	--	--	--	***c5***:**90**	0.9579	***c5***:**26**	0.9682	***c5***:**22**	0.9483	***c5***:**22**	0.9278
	***c5.300***:**100**	10.1	10±10	--	--	--	***c5***:**90**	0.9668	***c5***:**22**	0.9473	***c5***:**17**	0.9380	***c5***:**17**	0.9172
	***c5.300***:**132**	7.9	130±20	--	--	--	***c5***:**27**	0.8737	***c5***:**125**	0.7902	***c5***:**13**	0.8316	***c5***:**18**	0.8861
	***c5.300***:**140**	8.4	42±21	--	--	--	***c5***:**47**	0.9244	***c5***:**67**	0.9369	***c5***:**67**	0.9285	***c5***:**8**	0.8997
	***c5.300***:**147**	8.4	22±17	--	--	--	***c5***:**55**	0.9304	***c5***:**22**	0.9179	***c5***:**22**	0.9107	***c5***:**8**	0.9008
ILEmS	***c5.300***:**14**	11.4	13±12	--	***c5***:**38**	0.9402	--	--	***c5***:**26**	0.9369	***c5***:**22**	0.9345	***c5***:**22**	0.8979
	***c5.300***:**27**	10.4	67±27	--	***c5***:**19**	0.8877	--	--	***c5***:**22**	0.8734	***c5***:**22**	0.8820	***c5***:**22**	0.8619
	***c5.300***:**30**	9.5	5±4	--	***c5***:**34**	0.9337	--	--	***c5***:**67**	0.9161	***c5***:**67**	0.9000	***c5***:**8**	0.8995
	***c5.300***:**47**	14.3	11±16	--	***c5***:**34**	0.9625	--	--	***c5***:**67**	0.9776	***c5***:**67**	0.9755	***c5***:**8**	0.9318
	***c5.300***:**55**	8.6	30±26	--	***c5***:**147**	0.9304	--	--	***c5***:**22**	0.8931	***c5***:**22**	0.8869	***c5***:**18**	0.8703
	***c5.300***:**67**	14.3	25±22	--	***c5***:**38**	0.9569	--	--	***c5***:**17**	0.9419	***c5***:**22**	0.9323	***c5***:**22**	0.9173
	***c5.300***:**71**	1.0	153±0	--	***c5***:**132**	0.8339	--	--	***c5***:**125**	0.7300	***c5***:**27**	0.7912	***c5***:**18**	0.8923
	***c5.300***:**88**	8.6	23±26	--	***c5***:**34**	0.9215	--	--	***c5***:**23**	0.9380	***c5***:**23**	0.9300	***c5***:**8**	0.9055
	***c5.300***:**90**	21.9	3±3	--	***c5***:**100**	0.9668	--	--	***c5***:**17**	0.9614	***c5***:**17**	0.9578	***c5***:**17**	0.9357
ILEmL	***c5.300***:**6**	5.1	6±10	47±33	***c5***:**42**	0.9057	***c5***:**67**	0.9159	--	--	***c5***:**14**	0.9226	***c5***:**23**	0.9106
	***c5.300***:**13**	10.2	0±1	55±35	***c5***:**42**	0.9534	***c5***:**90**	0.9400	--	--	***c5***:**47**	0.9159	***c5***:**17**	0.9224
	***c5.300***:**17**	14.0	2±4	30±27	***c5***:**42**	0.9479	***c5***:**90**	0.9614	--	--	***c5***:**30**	0.9805	***c5***:**22**	0.9366
	***c5.300***:**22**	17.2	1±6	18±25	***c5***:**42**	0.9593	***c5***:**90**	0.9468	--	--	***c5***:**47**	0.9907	***c5***:**22**	0.9138
	***c5.300***:**23**	8.9	4±6	16±23	***c5***:**34**	0.9299	***c5***:**88**	0.9380	--	--	***c5***:**55**	0.9989	***c5***:**8**	0.9406
	***c5.300***:**26**	12.7	0±0	41±23	***c5***:**42**	0.9682	***c5***:**90**	0.9519	--	--	***c5***:**30**	0.9526	***c5***:**22**	0.9402
	***c5.300***:**67**	18.5	3±6	26±30	***c5***:**34**	0.9593	***c5***:**47**	0.9776	--	--	***c5***:**71**	0.9922	***c5***:**8**	0.9404
	***c5.300***:**125**	8.3	7±10	21±24	***c5***:**100**	0.9377	***c5***:**90**	0.9353	--	--	***c5***:**88**	0.9620	***c5***:**17**	0.9275
	***c5.300***:**141**	5.1	14±21	31±30	***c5***:**34**	0.9132	***c5***:**47**	0.9054	--	--	***c5***:**90**	0.9684	***c5***:**8**	0.8885
ILEmC	***c5.300***:**4**	24.0	13±5	--	***c5***:**42**	0.9100	***c5***:**90**	0.8957	***c5***:**26**	0.9186	***c5***:**17**	0.9022	--	--
	***c5.300***:**8**	12.0	3±2	--	***c5***:**34**	0.9343	***c5***:**47**	0.9318	***c5***:**23**	0.9406	***c5***:**67**	0.9348	--	--
	***c5.300***:**17**	20.0	4±4	--	***c5***:**42**	0.9275	***c5***:**90**	0.9357	***c5***:**26**	0.9320	***c5***:**125**	0.9078	--	--
	***c5.300***:**18**	16.0	3±5	--	***c5***:**100**	0.8861	***c5***:**71**	0.8923	***c5***:**26**	0.8861	***c5***:**26**	0.8908	--	--
	***c5.300***:**22**	16.0	3±2	--	***c5***:**42**	0.9278	***c5***:**67**	0.9173	***c5***:**26**	0.9402	***c5***:**17**	0.9226	--	--
	***c5.300***:**23**	12.0	9±6	--	***c5***:**42**	0.9011	***c5***:**90**	0.8941	***c5***:**125**	0.9234	***c5***:**125**	0.9132	--	--

^a^ Fraction of residues in cluster relative to all in ***chess square.parse***;

^b^ From GETAREA (Fraczkiewicz and Braun, 1998);

^c^ Adapted from GETAREA results as described in text;

^d^ Cluster map in ILE dataset most similar to cluster map named by row. Note that this may not be commutative;

^e^ Cluster map in ILEmS dataset most similar to cluster map named by row;

^f^ Cluster map in ILEmL dataset most similar to cluster map named by row;

^g^ Cluster map in ALAmN dataset most similar to cluster map named by row;

^h^ Cluster map in ALAmC dataset most similar to cluster map named by row.

#### Leucine

**FIGURE 10:**
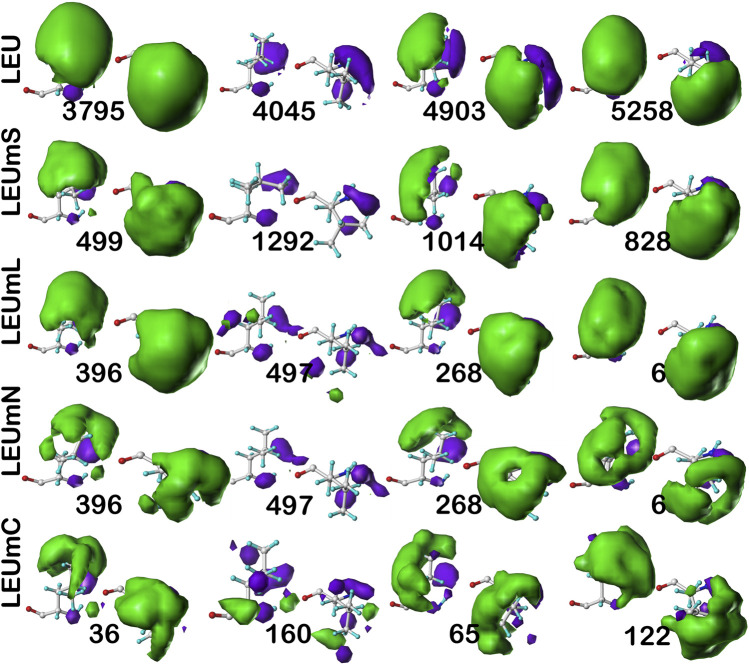
Three-dimensional clustered hydropathic interaction maps for leucine sidechains, in the ***c5*** chess square, χ_1_ = 300°. Each map pair (or cluster) is named by its “exemplar”, which is the number of the map, as defined in the text, closest to the cluster’s centroid. Top row – leucines from soluble proteins dataset; 2^nd^ row – leucines from soluble domains of membrane proteins dataset; 3^rd^ row – lipid-facing leucines in transmembrane domains, including residue-lipid interactions; 4^th^ row – as 3^rd^ row, ignoring residue-lipid interactions; and 5^th^ row – core-facing leucines in transmembrane domains. See also caption for Figure 6.

As above for isoleucine, the maps in Figure 10 illustrate four selected clusters of the ***c5.300*** cluster/parse of leucine. Table 5 lists the properties of all ***c5.300*** clusters for this residue (Supporting information Supplementary Tables S9A-E lists all residue and cluster data for the five leucine datasets and Supplementary Table S14 lists the similarity matrices for the ***b1*** and ***c5*** chess squares.). Interpretation of these maps and the associated cluster metrics is largely parallel to that of isoleucine. First, there are very obvious visual similarities in the LEU/LEUmS pairs displayed and the numerical data support these with three of the four >0.99. The solvent-exposed cluster pair **4045/1292** still has a strong similarity of almost 0.97. Also, both of these account for only ∼5% of their relative chess square/parse populations. Probably because leucine’s sidechain is more compact than that of isoleucine and it does not penetrate into the lipids as deeply, the differences between the LEUmL and LEUmN sets are not as clear as was seen in isoleucine maps. Non-etheless, each cluster map encodes this structural information. Remarkably, three of the four LEUmC maps (**36**, **65** and **122**, Figure 10; Table 4) have similarity metrics >0.97 to maps (**3795**, **4903** and **5258**, respectively) in the soluble protein LEU set, which is a reversal of observations made in isoleucine map comparisons; but, as mentioned above, this chess square and the *χ*_1_ = 300° parse is much more robustly populated in leucine, suggesting that the leucine results are perhaps more reliable, and that the transmembrane core residues actually *are* largely indistinguishable in terms of their interactions to residues in soluble proteins or domains. The large numbers of leucines seen in α helices, especially in interior locations of soluble proteins, and perhaps in the lipid-facing transmembrane regions, has been suggested to be an important factor in folding of α proteins (Nakashima et al., 2014).

**TABLE 5:** Cluster parameters and cluster-cluster similarities for leucine data sets.

	***Chess square.parse*:cluster**	Relative fraction^a^ (%)	SASA (Å^2^)^b^	LASA (Å^2^)^c^	Most similar LEU^d^		Most similar LEUmS^e^		Most similar LEUmL^f^		Most similar LEUmN^g^		Most similar LEUmC^h^	
					Cluster	metric	cluster	metric	cluster	Metric	cluster	metric	cluster	metric
LEU	***c5.300***:**997**	8.8	65±25	--	--	--	***c5*:1258**	0.9816	***c5*:568**	0.9661	***c5*:568**	0.9686	***c5*:36**	0.9232
	***c5.300***:**1645**	8.1	53±25	--	--	--	***c5*:1413**	0.9873	***c5*:968**	0.9696	***c5*:968**	0.9766	***c5*:122**	0.9463
	***c5.300***:**2101**	1.3	141±28	--	--	--	***c5*:1099**	0.9323	***c5*:497**	0.8257	***c5*:497**	0.8797	***c5*:160**	0.8192
	***c5.300***:**3795**	27.0	4±7	--	--	--	***c5*:499**	0.9930	***c5*:396**	0.9954	***c5*:396**	0.9887	***c5*:36**	0.9726
	***c5.300***:**4045**	4.8	107±23	--	--	--	***c5*:1292**	0.9686	***c5*:497**	0.8829	***c5*:497**	0.9361	***c5*:160**	0.8876
	***c5.300***:**4149**	16.0	28±20	--	--	--	***c5*:499**	0.9834	***c5*:268**	0.9924	***c5*:396**	0.9917	***c5*:36**	0.9741
	***c5.300***:**4885**	10.7	30±22	--	--	--	***c5*:828**	0.9858	***c5*:9**	0.9942	***c5*:9**	0.9913	***c5*:122**	0.9725
	***c5.300***:**4903**	9.7	10±13	--	--	--	***c5*:1014**	0.9910	***c5*:268**	0.9869	***c5*:268**	0.9835	***c5*:65**	0.9785
	***c5.300***:**5258**	13.5	7±10	--	--	--	***c5*:828**	0.9951	***c5*:6**	0.9884	***c5*:6**	0.9828	***c5*:122**	0.9866
LEUmS	***c5.300***:**499**	21.7	6±12	--	***c5*:3795**	0.9931	--	--	***c5*:396**	0.9888	***c5*:396**	0.9831	***c5*:36**	0.9768
	***c5.300***:**652**	5.5	35±22	--	***c5*:1645**	0.9213	--	--	***c5*:630**	0.9192	***c5*:630**	0.9316	***c5*:160**	0.9205
	***c5.300***:**828**	17.2	9±17	--	***c5*:5258**	0.9951	--	--	***c5*:9**	0.9926	***c5*:9**	0.9839	***c5*:122**	0.9891
	***c5.300***:**1014**	13.3	13±18	--	***c5*:4903**	0.9911	--	--	***c5*:268**	0.9861	***c5*:268**	0.9816	***c5*:75**	0.9838
	***c5.300***:**1099**	1.5	90±31	--	***c5*:2101**	0.9323	--	--	***c5*:497**	0.8288	***c5*:497**	0.8673	***c5*:160**	0.8274
	***c5.300***:**1258**	10.0	39±20	--	***c5*:997**	0.9816	--	--	***c5*:568**	0.9631	***c5*:568**	0.9599	***c5*:36**	0.9417
	***c5.300***:**1292**	5.5	70±22	--	***c5*:4045**	0.9686	--	--	***c5*:497**	0.8849	***c5*:497**	0.9520	***c5*:160**	0.8800
	***c5.300***:**1307**	12.7	18±15	--	***c5*:4885**	0.9856	--	--	***c5*:9**	0.9815	***c5*:9**	0.9835	***c5*:36**	0.9540
	***c5.300***:**1413**	12.8	16±18	--	***c5*:1645**	0.9873	--	--	***c5*:968**	0.9722	***c5*:968**	0.9651	***c5*:122**	0.9538
LEUmL	***c5.300***:**6**	17.9	1±4	24±27	***c5*:3795**	0.9925	***c5*:828**	0.9903	--	--	***c5*:6**	0.9979	***c5*:122**	0.9746
	***c5.300***:**9**	11.5	3±7	27±30	***c5*:4885**	0.9942	***c5*:828**	0.9926	--	--	***c5*:9**	0.9977	***c5*:122**	0.9848
	***c5.300***:**268**	10.6	3±8	25±29	***c5*:4149**	0.9924	***c5*:1014**	0.9861	--	--	***c5*:268**	0.9983	***c5*:36**	0.9754
	***c5.300***:**396**	15.3	2±5	24±25	***c5*:3795**	0.9954	***c5*:828**	0.9906	--	--	***c5*:396**	0.9966	***c5*:122**	0.9843
	***c5.300***:**497**	2.3	18±25	38±37	***c5*:1645**	0.8854	***c5*:1292**	0.8849	--	--	***c5*:497**	0.8983	***c5*:65**	0.8680
	***c5.300***:**568**	7.6	11±15	23±28	***c5*:4149**	0.9685	***c5*:1307**	0.9685	--	--	***c5*:568**	0.9952	***c5*:36**	0.9633
	***c5.300***:**630**	4.8	4±11	39±34	***c5*:4903**	0.9663	***c5*:1014**	0.9597	--	--	***c5*:630**	0.9934	***c5*:65**	0.9443
	***c5.300***:**968**	11.8	3±7	29±31	***c5*:5258**	0.9815	***c5*:828**	0.9851	--	--	***c5*:968**	0.9960	***c5*:122**	0.9714
	***c5.300***:**1139**	18.1	1±3	29±24	***c5*:3795**	0.9913	***c5*:828**	0.9885	--	--	***c5*:1139**	0.9949	***c5***:**122**	0.9747
LEUmC	***c5.300***:**10**	10.0	22±14	--	***c5*:4885**	0.9299	***c5*:1307**	0.9216	***c5*:9**	0.9417	***c5*:9**	0.9361	--	--
	***c5.300***:**36**	15.0	10±9	--	***c5*:3795**	0.9726	***c5*:499**	0.9768	***c5*:396**	0.9815	***c5*:396**	0.9749	--	--
	***c5.300***:**40**	5.0	48±11	--	***c5*:997**	0.9163	***c5*:1307**	0.9067	***c5*:568**	0.9013	***c5*:568**	0.9101	--	--
	***c5.300***:**56**	6.1	10±8	--	***c5*:4149**	0.9087	***c5*:1307**	0.9191	***c5*:396**	0.9220	***c5*:396**	0.9156	--	--
	***c5.300***:**65**	14.4	12±10	--	***c5*:4903**	0.9785	***c5*:1014**	0.9839	***c5*:268**	0.9717	***c5*:268**	0.9679	--	--
	***c5.300***:**75**	3.3	12±13	--	***c5*:5258**	0.9404	***c5*:1413**	0.9384	***c5*:6**	0.9479	***c5*:6**	0.9397	--	--
	***c5.300***:**122**	22.8	4±5	--	***c5*:5258**	0.9866	***c5*:828**	0.9892	***c5*:9**	0.9848	***c5*:9**	0.9755	--	--
	***c5.300***:**160**	8.3	36±21	--	***c5*:1645**	0.8895	***c5*:652**	0.9205	***c5*:630**	0.8843	***c5*:630**	0.8938	--	--
	***c5.300***:**176**	15.0	1±3	--	***c5*:5258**	0.9844	***c5*:828**	0.9811	***c5*:9**	0.9747	***c5*:9**	0.9669	--	--

^a^ Fraction of residues in cluster relative to all in *chess square.parse*;

^b^ From GETAREA (Fraczkiewicz and Braun, 1998);

^c^ Adapted from GETAREA results as described in text;

^d^ Cluster map in LEU dataset most similar to cluster map named by row. Note that this may not be commutative;

^e^ Cluster map in LEUmS dataset most similar to cluster map named by row;

^f^ Cluster map in LEUmL dataset most similar to cluster map named by row;

^g^ Cluster map in LEUmN dataset most similar to cluster map named by row;

^h^ Cluster map in LEUmC dataset most similar to cluster map named by row.

#### Proline

**FIGURE 11:**
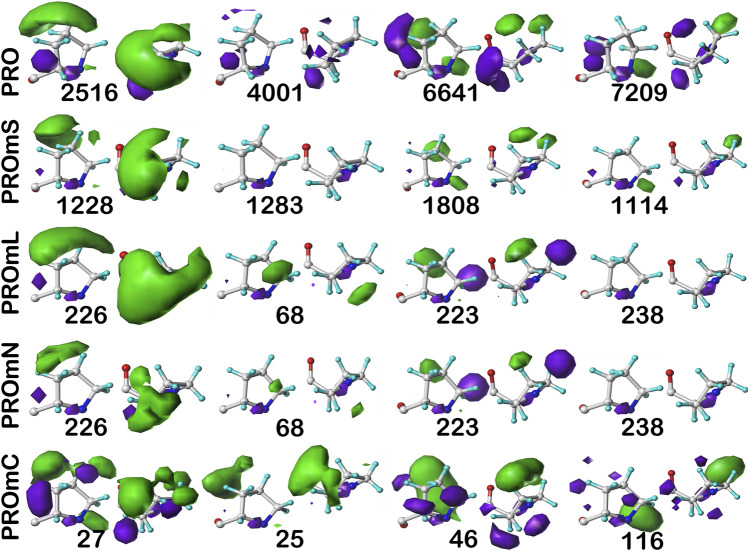
Three-dimensional clustered hydropathic interaction maps for proline sidechains, in the ***c8*** chess square, χ_1_ = 300°. Each map pair (or cluster) is named by its “exemplar”, which is the number of the map, as defined in the text, closest to the cluster’s centroid. Top row – prolines from soluble proteins dataset; 2^nd^ row – prolines from soluble domains of membrane proteins dataset; 3^rd^ row – lipid-facing prolines in transmembrane domains, including residue-lipid interactions; 4^th^ row – as 3^rd^ row, ignoring residue-lipid interactions; and 5^th^ row – core-facing prolines in transmembrane domains. See also caption for Figure 6.

The interaction maps for four clusters of the ***c8.30p*** prolines are displayed in Figure 11. In the nomenclature used to describe this conformation, it is in the polyproline II helical region. Because it is often termed a “helix breaker” residue, and helices comprise the large majority of secondary structure motifs seen in transmembrane regions, there are comparatively few prolines in the PROmL and PROmC datasets compared to the vast numbers of them in soluble proteins (PRO) and in the extramembrane (PROmS) domains of membrane proteins. Table 6 lays out numerical data describing the clustering of the ***c8.30p*** datasets (See also Supplementary Tables S10A-E and Supplementary Table S15 for more thorough data.) Prolines seem to be generally exposed: only one of the six clusters for ***c8.30p*** in PRO, **2516**, is dominated by hydrophobic interactions with its sidechain, which is also evident from its low SASA compared to the others. Perhaps this exposure is a cause or consequence of proline’s well-known role in disrupting helices in soluble proteins (Richardson and Richardson, 1988). Proline is less disruptive in transmembrane helices, generally inducing a kink (von Heijne, 1991; Wilman et al., 2014). Proline’s structural roles are environment-dependent (Li et al., 1996), but can be functional as well (Van Arnam et al., 2011). Very similar profiles and metrics are seen in the PROmS set. For prolines in the transmembrane region, their cyclic sidechains are not well-poised for deep penetration into the lipid: LEUmL’s average SASA, ∼7 Å^2^, is the largest of the hydrophobic residues of the lipid-facing RESmL datasets, while its LASA, ∼25 Å^2^, is not notably different from valine (23 Å^2^), isoleucine (25 Å^2^) or leucine (27 Å^2^). Other than for cluster **226**, the differences between the PROmL and PROmN maps are very minor. In contrast, cluster **68**, which has only modestly larger SASA, does not have seem to have significant residue-lipid interactions.

**TABLE 6:** Cluster parameters and cluster-cluster similarities for proline data sets.

	***Chess square.parse*:cluster**	Relative fraction^a^ (%)	SASA (Å^2^)^b^	LASA (Å^2^)^c^	Most similar PRO^d^		Most similar PROmS^e^		Most similar PROmL^f^		Most similar PROmN^g^		Most similar PROmC^h^	
					cluster	metric	cluster	Metric	cluster	metric	cluster	metric	cluster	metric
PRO	***c8.30p:*1297**	15.7	31±20	--	--	--	***c8:*110**	0.9557	***c8:*226**	0.9245	***c8:*226**	0.9323	***c8:*116**	0.9173
	***c8.30p:*1767**	6.5	93±18	--	--	--	***c8:*1057**	0.8822	***c8:*238**	0.8871	***c8:*238**	0.8869	***c8:*116**	0.8533
	***c8.30p:*2516**	30.5	5±8	--	--	--	***c8:*1228**	0.9879	***c8:*226**	0.9867	***c8:*124**	0.9783	***c8:*27**	0.9541
	***c8.30p:*4001**	11.2	43±26	--	--	--	***c8:*1283**	0.9497	***c8:*68**	0.9091	***c8:*68**	0.9103	***c8:*116**	0.8908
	***c8.30p:*6641**	19.0	44±21	--	--	--	***c8:*1808**	0.9697	***c8:*223**	0.9473	***c8:*74**	0.9470	***c8:*46**	0.9497
	***c8.30p:*7209**	17.1	81±19	--	--	--	***c8:*1114**	0.9251	***c8:*238**	0.8962	***c8:*74**	0.8947	***c8:*116**	0.9341
PROmS	***c8.30p:*110**	16.6	27±19	--	***c8:*1297**	0.9557	--	--	***c8:*226**	0.9334	***c8:*226**	0.9487	***c8:*25**	0.9336
	***c8.30p:*1057**	5.9	82±23	--	***c8:*1767**	0.8822	--	--	***c8:*238**	0.9151	***c8:*238**	0.9162	***c8:*116**	0.8358
	***c8.30p:*1114**	15.2	70±27	--	***c8:*7209**	0.9251	--	--	***c8:*238**	0.9498	***c8:*238**	0.9526	***c8:*116**	0.9278
	***c8.30p:*1228**	30.2	5±9	--	***c8:*2516**	0.9879	--	--	***c8:*226**	0.9881	***c8:*226**	0.9857	***c8:*27**	0.9584
	***c8.30p:*1283**	12.4	33±22	--	***c8:*4001**	0.9497	--	--	***c8:*68**	0.9250	***c8:*226**	0.9279	***c8:*56**	0.8993
	***c8.30p:*1808**	19.6	37±23	--	***c8:*6641**	0.9697	--	--	***c8:*223**	0.9627	***c8:*74**	0.9747	***c8:*46**	0.9352
PROmL	***c8.30p:*68**	16.5	6±14	44±32	***c8:*2516**	0.9538	***c8:*1228**	0.9632	--	--	***c8:*68**	0.9639	***c8:*56**	0.9222
	***c8.30p:*74**	18.9	4±7	32±34	***c8:*2516**	0.9425	***c8:*1228**	0.9550	--	--	***c8:*74**	0.9769	***c8:*27**	0.9468
	***c8.30p:*124**	21.8	3±7	17±25	***c8:*2516**	0.9847	***c8:*1228**	0.9874	--	--	***c8:*124**	0.9982	***c8:*27**	0.9647
	***c8.30p:*223**	17.4	19±23	30±33	***c8:*6641**	0.9473	***c8:*1808**	0.9627	--	--	***c8:*223**	0.9966	***c8:*46**	0.9393
	***c8.30p:*226**	17.4	3±6	24±33	***c8:*2516**	0.9867	***c8:*1228**	0.9881	--	--	***c8:*226**	0.9920	***c8:*27**	0.9591
	***c8.30p:*238**	8.0	38±43	43±42	***c8:*7209**	0.8963	***c8:*1114**	0.9498	--	--	***c8:*238**	0.9930	***c8:*116**	0.9016
PROmC	***c8.30p:*25**	19.7	17±16	--	***c8:*2516**	0.9397	***c8:*1228**	0.9363	***c8:*124**	0.9346	***c8:*226**	0.9408	--	--
	***c8.30p:*27**	37.6	4±7	--	***c8:*2516**	0.9541	***c8:*1228**	0.9584	***c8:*124**	0.9647	***c8:*124**	0.9646	--	--
	***c8.30p:*46**	7.7	20±11	--	***c8:*6641**	0.9497	***c8:*1808**	0.9352	***c8:*223**	0.9393	***c8:*223**	0.9260	--	--
	***c8.30p:*56**	17.9	8±7	--	***c8:*2516**	0.9319	***c8:*1228**	0.9469	***c8:*124**	0.9439	***c8:*124**	0.9384	--	--
	***c8.30p:88***	6.0	19±11	--	***c8:*1297**	0.8895	***c8:*110**	0.8952	***c8:*124**	0.8827	***c8:*124**	0.8853	--	--
	***c8.30p:*116**	11.1	29±21	--	***c8:*7209**	0.9341	***c8:*1114**	0.9279	***c8:*238**	0.9016	***c8:*74**	0.9142	--	--

^a^ Fraction of residues in cluster relative to all in *chess square. parse*;

^b^ From GETAREA (Fraczkiewicz and Braun, 1998);

^c^ Adapted from GETAREA results as described in text;

^d^ Cluster map in PRO dataset most similar to cluster map named by row. Note that this may not be commutative;

^e^ Cluster map in PROmS dataset most similar to cluster map named by row;

^f^ Cluster map in PROmL dataset most similar to cluster map named by row;

^g^ Cluster map in PROmN dataset most similar to cluster map named by row;

^h^ Cluster map in PROmC dataset most similar to cluster map named by row.

### Interaction character and accessibility

**FIGURE 12:**
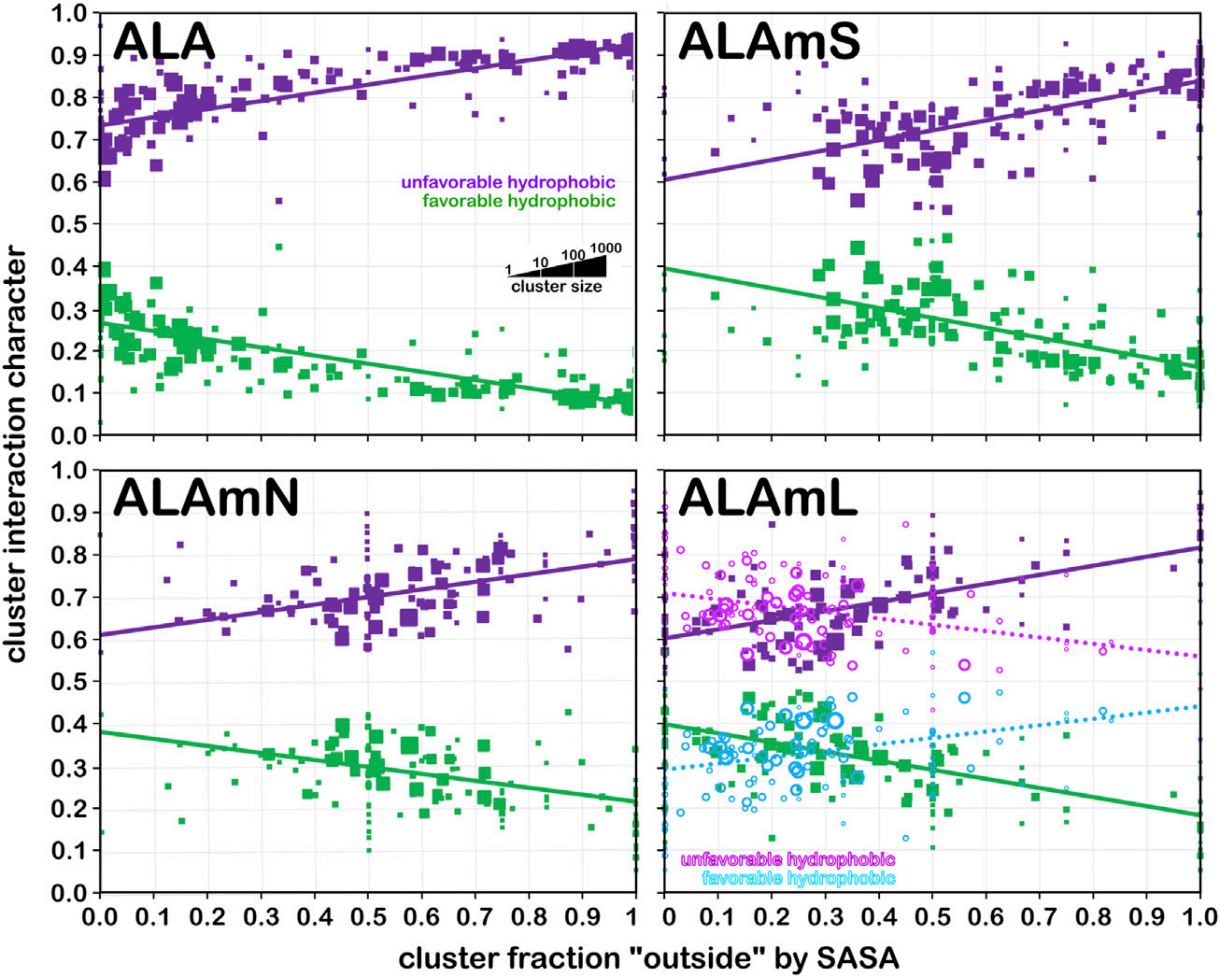
Interaction character as a function of residue accessibility for alanine datasets. Each data marker represents a cluster whose size is scaled by population of its associated cluster; fit lines are from weighted (by population) least squares. Green markers and fit lines represent favorable hydrophobic fraction of interaction character and purple markers and fit lines represent unfavorable hydrophobic fraction of interaction character when accessibility is SASA; cyan and magenta markers and fit lines show character when accessibility is LASA. See text for further description of results.

We showed in earlier work (AL Mughram et al., 2021a; Herrington and Kellogg, 2021; Catalano et al., 2021) that plotting interaction character as a function of our derived solvent-accessible surface area metric, *f*_*outside*_, was useful for understanding residue roles in structure. Figure 12 presents that analysis for i) alanine from the soluble dataset (ALA); ii) alanine from the soluble domain(s) for the membrane dataset; iii) alanine from the lipid-facing dataset where lipid-residue interactions were not calculated (ALAmN); and iv) alanine with lipid interactions included (ALAmL) and the accessibility plotted as f_outside_ (SASA) and f_outside_ (LASA). Unsurprisingly, in the soluble proteins alanine dataset (Figure 12, upper left), as accessibility increases, interaction character shifts from ∼30% hydrophobic at *f*_*outside*_ near zero to ∼10% hydrophobic at full exposure (*f*_*outside*_ = 1). The trends in the ALAmS dataset are similar (Figure 12, upper right), although there are significantly fewer clusters at small values of *f*_*outside*_, and the slopes of the population-weighted fit lines are more aggressive. In Figure 12, lower left, the largest portion of the data is 0.35 < f_outside_ < 0.75, which suggests that many alanines in the lipid-facing transmembrane region are more involved with interactions within their (largely helical) domains than externally. To further explore these structural concepts, *f*_*outside*_, calculated with interactions between the alanines and artificial lipids, was decomposed into its “solvent” and “lipid” accessible portions, as displayed in Figure 12, lower right. Here it can be seen that increased lipid accessibility does appear to lead to a larger hydrophobic interaction character, but it should be stated that there is very little data past the 50% accessible level. ALAmC (data not shown) is largely consistent with ALA.

**FIGURE 13:**
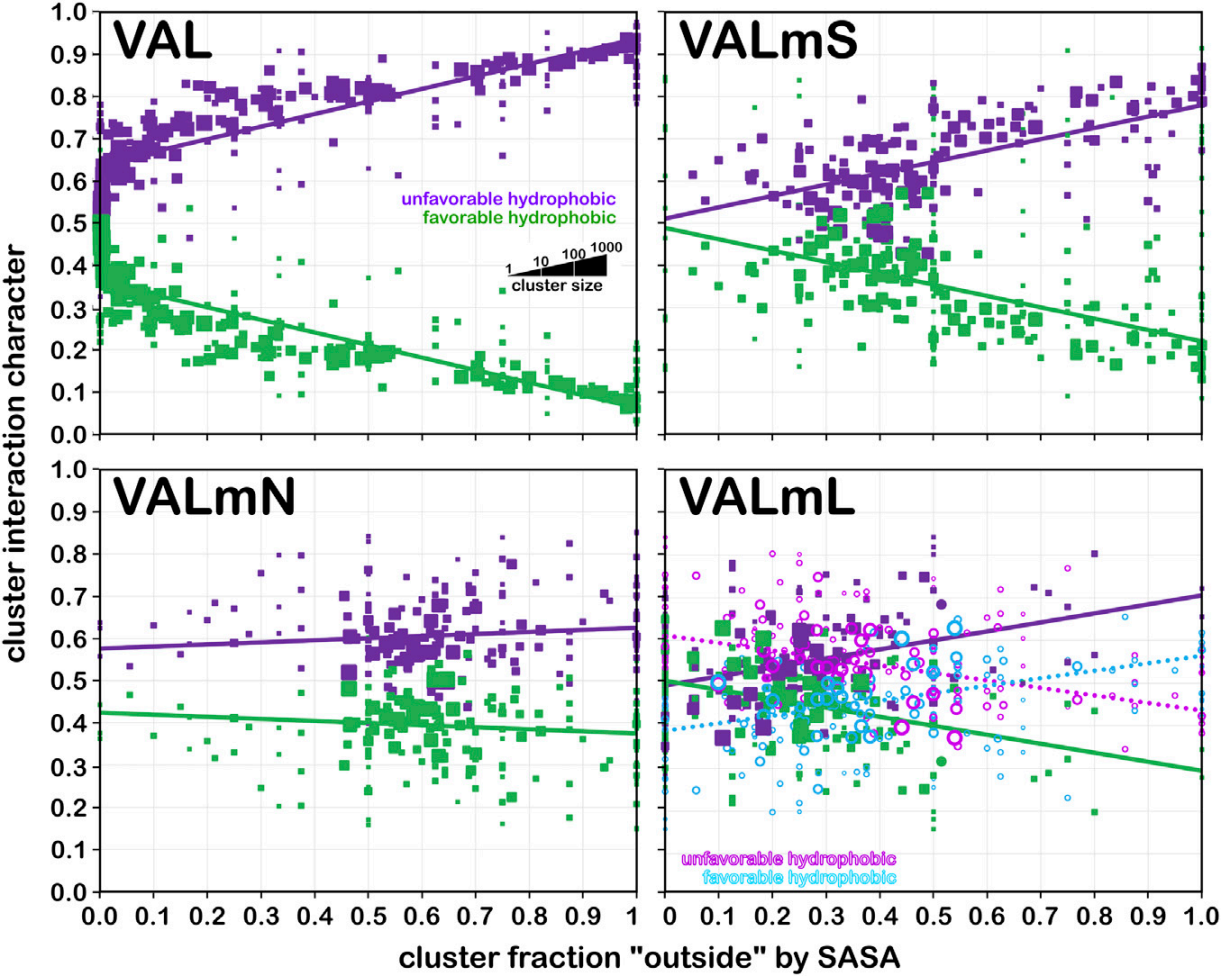
Interaction character as a function of residue accessibility for valine datasets. See also caption for Figure 12.

Valine is a somewhat larger hydrophobic residue than alanine. Figure 13 displays the same set of plots for this residue. Because of its more hydrophobic nature, it has both a higher fraction of hydrophobic interactions at low *f*_*outside*_, but that drops more rapidly as *f*_*outside*_ approaches one compared to alanine (Figure 13, upper left). Trends similar to those of alanine in the other three quadrants of Figure 13, modified by valine’s larger size and hydrophobicity, are seen.

**FIGURE 14:**
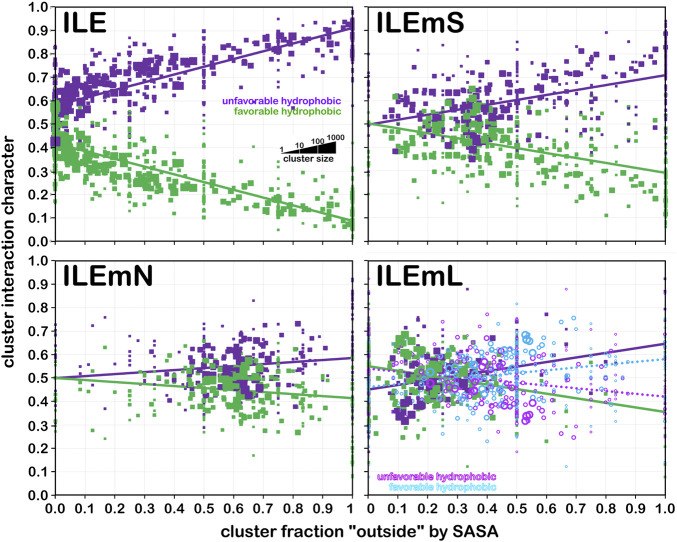
Interaction character as a function of residue accessibility for isoleucine datasets. See also caption for Figure 12.

Larger hydrophobic residues, such as isoleucine (Figure 14) have, as expected, more hydrophobic interactions. In fact, at *f*_*outside*_ near zero, interactions are almost exactly half hydrophobic and half hydrophobic-polar. In the soluble dataset (Figure 14, upper left), as exposure increases—very likely to water, the fraction of hydrophobic interactions drops precipitously. In contrast, in the soluble domain of membrane proteins (ILEmS, Figure 14, upper right), that drop is less dramatic and possesses a slope similar to the analogous alanine plot. There are, more, however, lower-valued *f*_*outside*_ clusters than in alanine. ILEmN (Figure 14, lower left) shows narrow range of highly populated clusters: 0.45 < *f*_*outside*_ < 0.85, and a weak dependence on *f*_*outside*_. Since this dataset does not include interactions with the lipids, and there are no water molecules in the membrane protein models, the observed interactions are wholly associated with the residue-residue sidechain interactions in the protein itself, and suggest a delicate balance of hydrophobic and polar residues in this region of a membrane protein. This balance is manifested with more hydrophobic residues (isoleucine, leucine, proline and valine, with methionine, phenylalanine also being more prevalent), and a stronger tendency for the smaller (glycine, serine and threonine) over the longer chain polar residues (Eilers et al., 2002; Jaakola et al., 2005; Baeza-Delgado et al., 2013). Also, the DeGrado group and others have analyzed helix-helix interactions and packing in numerous studies (Eilers et al., 2002; Gimpelev et al., 2004; Walters and DeGrado, 2006; Zhang et al., 2009; Zhang et al., 2015) that are largely supportive of our observations. Leucine data for this analysis is very similar, but available in supporting information as Supplementary Figure S3. In the same way, the data for proline is more or less the same as valine, but available as Supplementary Figure S4.

## Summary and conclusion

This study had a number of objectives. First, we wished to characterize the residue interaction environments of the hydrophobic residues, alanine, isoleucine, leucine, proline and valine to complement our earlier studies of the aromatic residues (phenylalanine, tyrosine and tryptophan) (AL Mughram et al., 2021a), the ionizable residues (aspartic acid, glutamic acid and histidine) (Herrington and Kellogg, 2021) and the isostructural residues serine and cysteine (Catalano et al., 2021). The latter work also explored, for the first time with our approach, the differences between soluble proteins and membrane proteins. That analysis, although revealing, was somewhat limited because no distinction was made amongst the multiple potential structural domains of membrane proteins. The second objective of this work, thus, was to identify broad classes of residues that performed unique structural roles in membrane proteins, and characterize these residues in terms of their interaction environments and other properties. Here, we used concepts and parameters described in the MemProtMD database (Newport et al., 2019) to define membrane protein residue sets that are: 1) in soluble domains, 2) transmembrane and facing the lipids, and 3) transmembrane and facing the core. Lastly, we are continuing to assess the value of this map paradigm in protein structure prediction scenarios.

The 3D maps we calculated illustrate the type, strength and spatial location of interactions between the residue of interest and all surrounding residues and water (if present). While each residue in each protein is, of course, unique, we have shown through this and previous studies that encoding their interactions in 3D maps binned by backbone angles and (when necessary) χ_1_, followed by clustering and intra-cluster averaging, reveals a much more limited set of maps. For the hydrophobic residues, the interaction types are limited to favorable and unfavorable hydrophobic. Their profiles reveal the specific character and loci of their interacting partners. Our 2019 report (Ahmed et al.) showed that these maps are, in effect, a reproducible motif of structure because similar backbone angle bins yielded maps that were both visually and numerically very similar. Also seen was that the solvent-accessible surface areas (SASAs) of highly similar maps are also the same. The present study confirms this assertion in an emphatic manner. We found that even maps from unique and distinct datasets also often had very high similarities, even remarkably so.

Although we expected that residues in soluble proteins and in the soluble domains of membrane proteins would likely be similar, the fact that their interaction maps were often indistinguishable was surprising. We did note that the SASAs for the latter cases appeared to be somewhat larger, which we hypothesize may be, at least in part, an artifact of the conditions required to crystallize membrane proteins. While commonalities in interaction environments exist between residues in soluble proteins and in the lipid-facing transmembrane domain, there are differences as well. Treating them as unique data sets allows for more nuanced analyses, such as exploring and isolating the specific features due to residue-lipid interactions. These features are the nodes of a three-dimensional network where each residue map is a puzzle piece. It is surprising, however, that these “inside-out” residues where the solvent is a lipid are even remotely similar in terms of their interactions with environment. The numbers of residues falling in the last category—“core” transmembrane—is unfortunately small, less than 5% of those in the soluble protein set. Thus, clustering is less precise, and the ensuing calculations are more uncertain. Nevertheless, the RESmC maps are more than broadly similar to the other sets.

In addition to the residue types that we have analyzed here, and in our previous reports, we have now completed most calculations for all residue types. While there are certainly other interesting stories to relate concerning these residues, our more immediate goal is to apply these maps and associated metrics in building three-dimensional protein structure models. With the new knowledge gained for membrane proteins related in this article, we believe that our approach—incorporating indirect structural effects like the pi-pi stacking and pi-cation interactions of aromatic residues (AL Mughram et al., 2021a), the role of ionization states in structure for ionizable residues (Herrington and Kellogg, 2021), the differences between residues in soluble and membrane proteins as in this work, and our generally robust and rational treatment of hydrophobic interactions—has significant promise. We term our methods “3D interaction homology” because the maps are agnostic with respect to the identity of neighboring (environment) residues, but are instead focused on the three-dimensional arrangement of interactions and their types. This is a fundamental difference from *de novo* structure prediction tools like AlphaFold (Senior et al., 2019; Senior et al., 2020), Rosetta (Barth et al., 2009; Yang et al., 2020), and the newly reported ESMFold (Callaway, 2022), which largely base their predictions on sequence homology. Lower-level predictions such as rotamer conformation, *etc.*, are not handled very well in these methods, likely to the extent that such predicted structures will be inadequate for drug discovery applications where sidechain orientations are critical. Rotamer library-based methods (Ponder and Richards, 1987; Headd et al., 2009; Bhuyan and Gao, 2011; Scouras and Daggett, 2011), such as SCWRL (Bower et al., 1997; Wang et al., 2008; Krivov et al., 2009) do fill in a lot of such gaps but are seemingly lacking in providing an understanding of structure. Our paradigm is another way to approach this information gap in numerous applications such as protein-protein docking, optimizing sidechains after site-directed mutagenesis or low-to-medium density residue replacement in homology-built models, or after *de novo* folding. Lastly, this may be an especially relevant approach for building better membrane protein models where native or even reasonably similar lipids are rarely present in the crystals or cryo-EM particles, and misinterpretations of reported structures have been published (Rawson et al., 2016; Guo, 2020; Yao et al., 2020; Ravikumar et al., 2021).

## Data Availability

The datasets presented in this study can be found in online repositories. The names of the repository/repositories and accession number(s) can be found in the article/Supplementary Material.
