# Supplementary material for: 3D interaction homology: The hydrophobic residues alanine, isoleucine, leucine, proline and valine play different structural roles in soluble and membrane proteins
Source: Front Mol Biosci. 2023 Mar 28;10:1116868. doi: 10.3389/fmolb.2023.1116868 (PMC10086146; doi:10.3389/fmolb.2023.1116868)
Supplement: Supplementary file 1 [file DataSheet1.zip › AL_Mughram_et_al_SupportingInformation/FiguresS1-S4.pdf]

3D Interaction Homology: The Hydrophobic Residues Alanine, Isoleucine, Leucine, Proline and Valine Play Different Structural Roles in Soluble and Membrane Proteins.

Mohammed H. AL Mughram, Claudio Catalano, Noah B. Herrington, Martin K. Safo and Glen E. Kellogg*

***Correspondence**: Glen E. Kellogg, glen.kellogg@vcu.edu

Figure S1: Population and solvent accessibility plots for leucine by chess square

Figure S2: Population and solvent accessibility plots for valine by chess square

Figure S3: Interaction character as a function of residue accessibility for leucine datasets.

Figure S4: Interaction character as a function of residue accessibility for proline datasets.

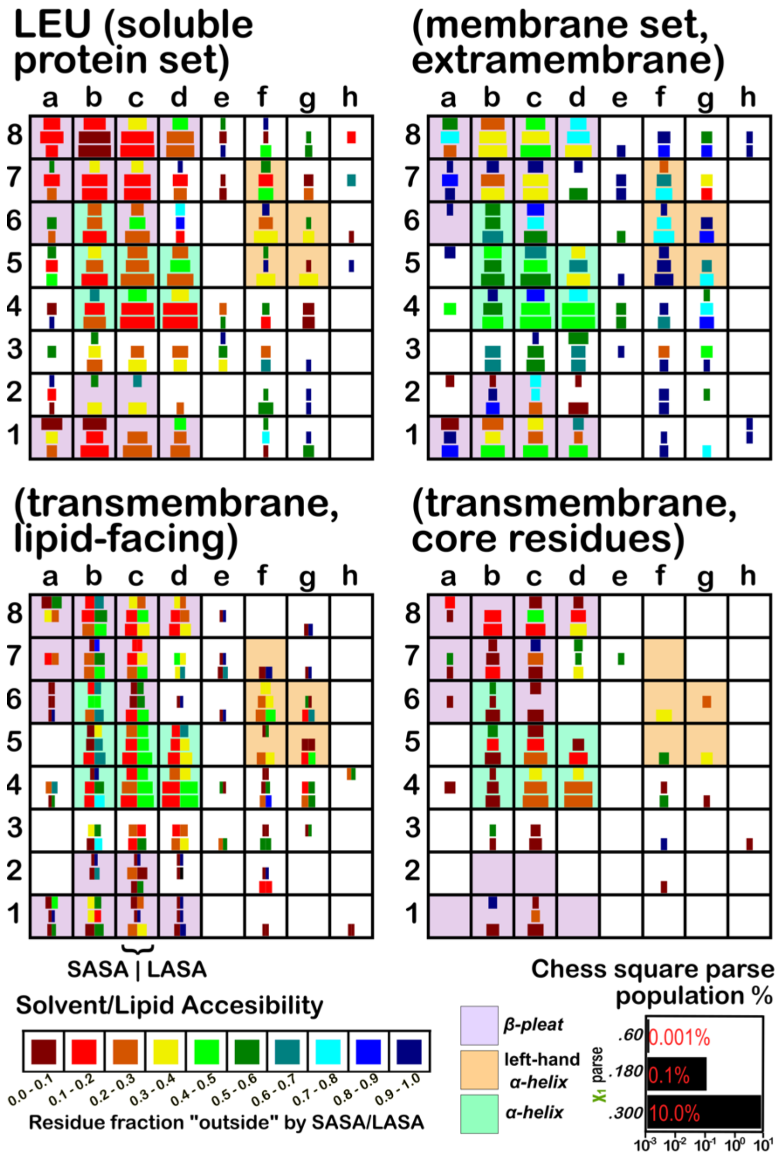

Figure S1: Population and solvent accessibility plots for leucine by chess square. Bar lengths are logarithmically proportional to population, bar colors encode the fraction of residues in that chess square exposed to solvent or lipid, as defined by the inset color map. Background colors show secondary structure.

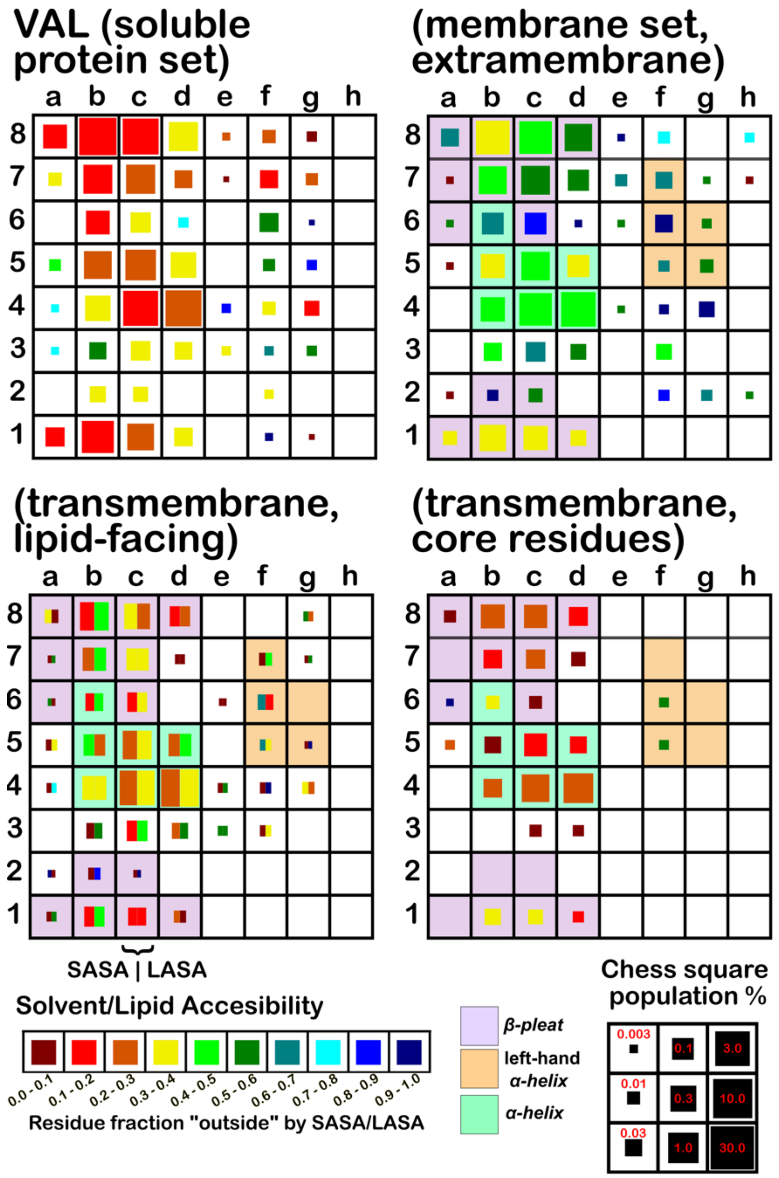

Figure S2: Population and solvent accessibility plots for valine by chess square. Square sizes are logarithmically proportional to population, square colors encode the fraction of residues in that chess square exposed to solvent or lipid, as defined by the inset color map. Background colors show secondary structure.

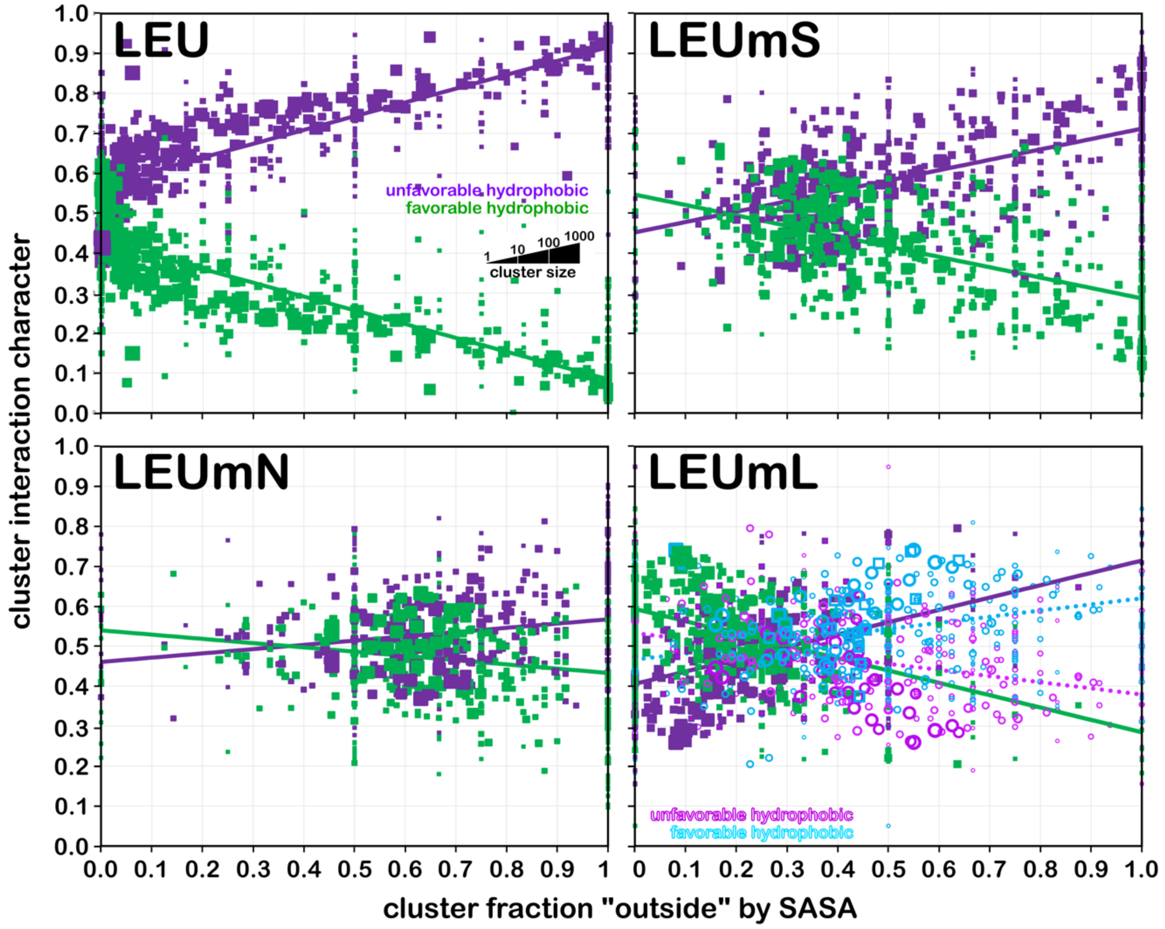

Figure S3: Interaction character as a function of residue accessibility for leucine datasets. Each data marker represents a cluster whose size is scaled by population of its associated cluster; fit lines are from weighted (by population) least squares. Green markers and fit lines represent favorable hydrophobic fraction of interaction character and purple markers and fit lines represent unfavorable hydrophobic fraction of interaction character when accessibility is SASA; cyan and magenta markers and fit lines show character when accessibility is LASA. See text for further description of results.

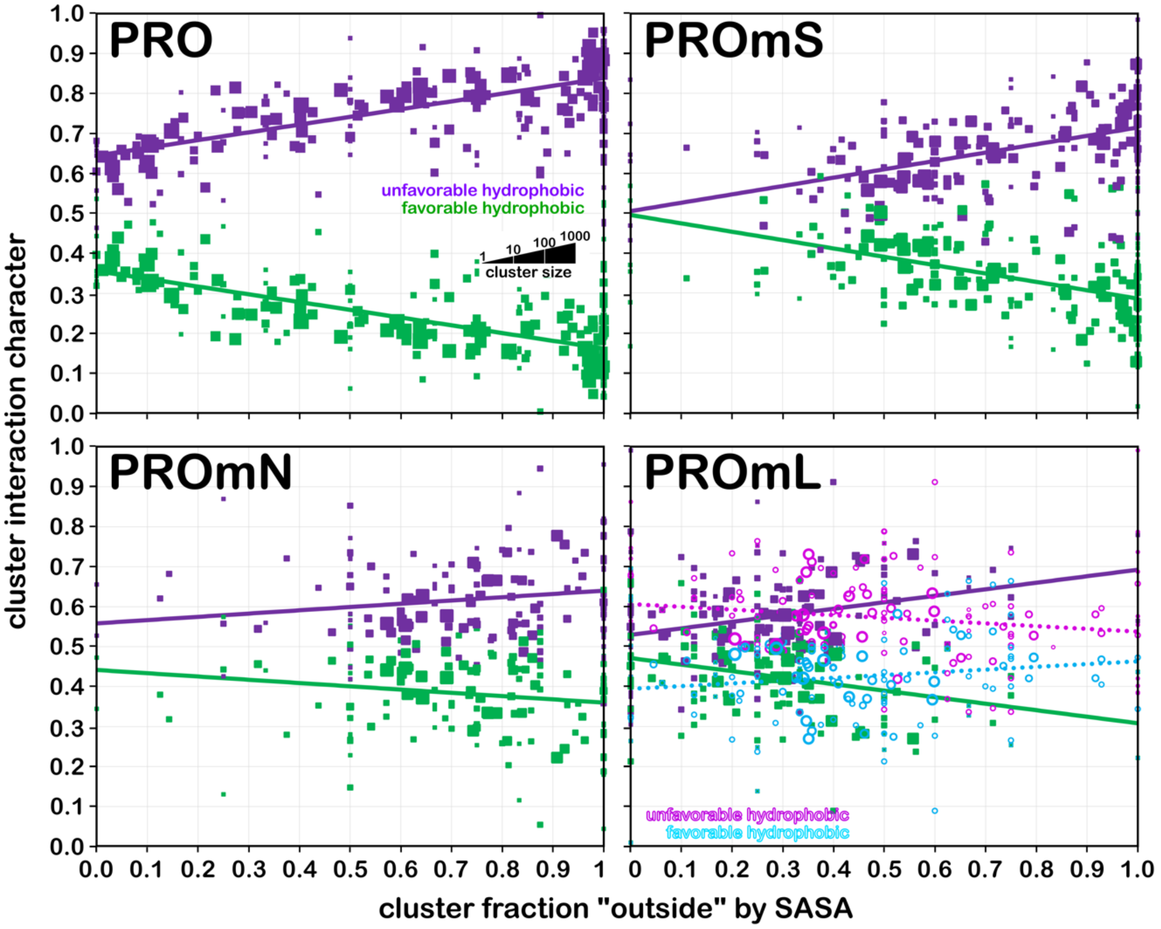

Figure S4: Interaction character as a function of residue accessibility for proline datasets. Each data marker represents a cluster whose size is scaled by population of its associated cluster; fit lines are from weighted (by population) least squares. Green markers and fit lines represent favorable hydrophobic fraction of interaction character and purple markers and fit lines represent unfavorable hydrophobic fraction of interaction character when accessibility is SASA; cyan and magenta markers and fit lines show character when accessibility is LASA. See text for further description of results.
